# Fluorene-Based Donor-Acceptor Copolymers Containing Functionalized Benzotriazole Units: Tunable Emission and their Electrical Properties †

**DOI:** 10.3390/polym12020256

**Published:** 2020-01-22

**Authors:** Iván Torres-Moya, Rebeca Vázquez-Guilló, Sara Fernández-Palacios, José Ramón Carrillo, Ángel Díaz-Ortiz, Juan Teodomiro López Navarrete, Rocío Ponce Ortiz, Mari Carmen Ruiz Delgado, Ricardo Mallavia, Pilar Prieto

**Affiliations:** 1 Department of Organic Chemistry, Faculty of Chemical Sciences and Technologies-IRICA, University of Castilla-La Mancha, 13071 Ciudad Real, Spain; Ivan.TorresMoya@uclm.es (I.T.-M.); Joseramon.carrillo@uclm.es (J.R.C.); angel.diaz@uclm.es (Á.D.-O.); 2 Instituto de Investigación Desarrollo e innovación en Biotecnología Sanitaria de Elche (IDiBE), University of Miguel Hernández, 03202 Elche, Spain; rebecavg73@gmail.com; 3 Department of Physical Chemistry, Faculty of Sciences, University of Málaga, 29071 Málaga, Spain; sarafpc@uma.es (S.F.-P.); teodomiro@uma.es (J.T.L.N.); rocioponce@uma.es (R.P.O.)

**Keywords:** synthesis, benzotriazole, fluorene, oligomers, copolymers, tunable emission, OFETs

## Abstract

Monomers 4,7-dibromo-2*H*-benzo[*d*]1,2,3-triazole (**m1**) and 4,7-(bis(*4*-bromophenyl)ethynyl)-2*H*-benzo[*d*]1,2,3-triazole (**m2**) have been synthesized in good yields using different procedures. Monomers **m1** and **m2** have been employed for building new copolymers of fluorene derivatives by a Suzuki reaction under microwave irradiation using the same conditions. In each case different chain lengths have been achieved, while **m1** gives rise to polymers for **m2** oligomers have been obtained (with a number of monomer units lower than 7). Special interest has been paid to their photophysical properties due to excited state properties of these D-A units alternates, which have been investigated by density functional theory (DFT) calculations using two methods: (i) An oligomer approach and (ii) by periodic boundary conditions (PBC). It is highly remarkable the tunability of the photophysical properties as a function of the different monomer functionalization derived from 2*H*-benzo[*d*]1,2,3-triazole units. In fact, a strong modulation of the absorption and emission properties have been found by functionalizing the nitrogen N-2 of the benzotriazole units or by elongation of the π-conjugated core with the introduction of alkynylphenyl groups. Furthermore, the charge transport properties of these newly synthesized macromolecules have been approached by their implementation in organic field-effect transistors (OFETs) in order to assess their potential as active materials in organic optoelectronics.

## 1. Introduction

The semiconductive nature of conjugated polymers makes them attractive candidates as alternatives to inorganic materials in electronics and photonics, principally due to their promise for low cost and large-scale processing of devices such as light-emitting diodes [1], solar cells [2,3,4,5,6], and organic field-effect transistors (OFETs) [7,8,9,10,11,12]. In addition, synthetic chemistry is a powerful tool to create new routes for the design and synthesis of new conjugated materials and device architectures, allowing modification of chemical structures and the development of new polymeric formulations, thus allowing the control of their optical and electronic properties. Molecular and device designs inspired in modified polymers, either at the backbone or at lateral chains, have been used to achieve blue or red-shifted absorptions, water solubility, photo and electroluminescence, interaction or coupling to active agents, etc. [13,14,15,16]. All of these facts lead to the achievement of higher performances and more stable devices.

Among the possible synthetic tools, modulation of conjugation length is feasible by modifying the degree of coplanarity in the polymer backbone. In the same way, the introduction of acceptor (A) and donor groups (D) or extending the conjugation causes hypsochromic or bathochromic shifts in luminescence spectrum [17]. One of the best, easiest, and most effective approaches to extend the absorption range is to design random D-A copolymers consisting of three or more monomers with complementary absorptions or the blending of two or more different polymers containing mono-acceptor and mono-donor with complementary absorption [18,19,20,21]. For example, full color displays can be formulated using classical structures like homopoly(9,9-di-n-octylfluoren-2,7-diyl) (PFO, λ_emax_ = 417 nm), poly[(9,9-di-n-octylfluoren-2,7-diyl)-*alt*-(benzo[2,1,3]thiadiazol-4,8-diyl)] (F8BT, λ_emax_ = 515 nm), and poly[2-methoxy-5-(2-ethylhexyloxy)-*alt*-1,4-phenylenevinylene] (MEH-PPV, λ_emax_ = 554 nm) due to the variety of band gaps that can be realized through chemical modification.

The variation of the Highest Occupied Molecular Orbital (HOMO) and the Lowest Unoccupied Molecular Orbital (LUMO) energy levels by a synthetic approach is also interesting to modulate the semiconductor ability to act as p-type, n-type, or ambipolar semiconductors [22,23,24]. Due to its easy modification, benzotriazole unit has been widely used as an electron-acceptor unit to build medium or wide band-gap D-A conjugated polymers in organic electronics [25,26,27]. Recently, dramatic development has been achieved in organic solar cells and in other electronic devices by using the benzotriazole-based polymers as the electron acceptor with small band-gap donor groups [28].

On the other hand, benzotriazole unit is a multitask skeleton which has been used in other areas, for example, as organic waveguides [29], in organogels [30], as semiconductors in OFETs [31], and as fluorescence sensors in biomedicine [32]. Previously in our groups, we have described the use of 4,7-dialkynyl-2*H*-benzo[*d*]1,2,3-triazole derivatives as semiconductors in OFETs [31].

**Scheme 1:**
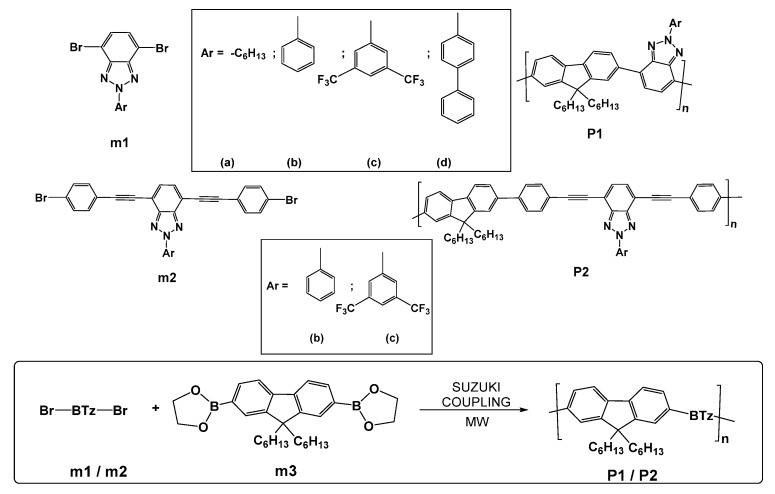
General scheme for the synthesis of **P1** and **P2** derived from 2*H*-benzo[*d*]1,2,3-triazoles by a Suzuki polycondensation reaction between dibromo benzotriazole derivatives **m1**, **m2**, and 9,9-dihexylfluorene-2,7-diboronic acid bis(1,3-propanediol) ester (**m3**).

In this work, we propose a series of macromolecules based on fluorene and different 2*H*-benzo[*d*]1,2,3-triazole derivatives with four different groups in order to modulate their acceptor character, aiming at studying their photophysical properties and improving the semiconductor character of the alkynyl-2*H*-benzo[*d*]1,2,3-triazole moieties (Scheme 1) for applications in optoelectronics.

## 2. Materials and Methods

Reagents were used as purchased. All the reactions which are air-sensitive were carried out under an argon atmosphere. A CEM (Matthews, NC, USA) Discover microwave reactor with magnetic stirring was used for the synthesis under microwave irradiation. This equipment comprises a pressure sensor (IntelliVent) and an infrared (IR) sensor allows to monitor and control the temperature. The reactions were performed in standard Pyrex^®^ tubes of 10 mL. The method employed was SPS (solid phase synthesis) with a pulse of 150 W applied maintaining the sample at a temperature of 135 ± 5 °C for 22 min.

A Varian Unity 500 (^1^H: 500 MHz; ^13^C: 125 MHz) spectrometer (Illinois, US) at 298 K was employed to record the NMR spectra employing deuterated solvents and all the signals were internally referenced against the TMS (trimethylsilane) solvent signal. Coupling constants (J) are denoted in Hz and chemical shifts (δ) in ppm. Multiplicities are denoted as follows: s = singlet, d = doublet, t = triplet, m = multiplet, brs = broad singlet. A Varian Cary model 5000 UV-Vis-NIR spectrophotometer (Agilent, Santa Clara, US) was employed to record the UV-vis spectra, using standard quartz cells of 1 cm width and solvents of high spectroscopic grade. IR spectra were registered on an IR Shimadzu, FTIR Affinity 1S WL C/Labsolution system (Shimadzu, Tokio, Japón), with a zinc selenide crystal and ATR device. An FT Raman accessory kit (RamIl) on a Bruker Vertex 70 FT-IR interferometer (Bruker, Billerica, US) and a continuous-wave Nd:YAG laser were employed to perform the FT-Raman spectra with an excitation at 1064 nm. In addition, a germanium detector operating at liquid-nitrogen temperature was performed and the Raman scattering radiation was collected in a back-scattering configuration with a 4 cm^−1^ spectral resolution. In the reported spectra, an average of 1000 scans was employed.

Size exclusion chromatographic (SEC) analysis was performed on a Shimadzu LC-20AD (Shimadzu, Tokio, Japón), index refraction detector RID-10A (Shimadzu, Kyoto, Japón) and on an evaporative light scattering detector (ELSD 3300, Alltech Associates, Inc., (Deerfield, IL, USA). The procedure employed was the following one: 20 μL of samples was injected on a column PLgel 5 μm MIXED-C; 2 × (300 × 7.5 mm) from Polymer Laboratories Ltd. (Salop, UK). Between 3–5 mg of each sample was dissolved in 1 mL of THF (as eluent) and were filtered through a 0.45 μm nylon syringe filter. In order to calculate the number-average (*M_n_*) and weight-average (*M_w_*) molecular weights Polymer Laboratories EasiCal Polystyrene standards for an accurate calibration were used.

Synthesis of monomers:

*4,7-dibromo-2-hexyl-2H-benzo[d]1,2,3-triazole* (**m1a**): Bromination of benzotriazole **2** (5 g, 42.02 mmol) employing the procedure described by Ekiz [33] afforded a transparent liquid identified as **m1a** (6.23 g, 73%). ^1^H-NMR (CDCl_3_, ppm) δ: 7.48 (s, 2H, H_BTz_), 4.83–4.80 (t, 2H, *J* = 7.4 Hz, N-CH_2_), 2.18–2.17 (t, 2H, *J* = 7.4 Hz, –CH_2_), 1.37–1.35 (m, 6H, 3 × –CH_2_), 0.93–0.91 (t, 3H, *J* = 6.8 Hz, –CH_3_). ^13^C-NMR (CDCl_3_, ppm) δ: 143.7, 129.5, 110.0, 57.5, 31.1, 31.0, 26.2, 22.4, 13.9. IR (neat, cm^−1^): 2939.76, 1462.35, 1321.54, 741.72, 670.93. MS calculated for (C_12_H_15_Br_2_N_3_) M^+^ 358.96, found 359.15. Anal. calcd. for (C_12_H_15_Br_2_N_3_): C, 39.92; H, 4.19; Br, 44.26; N, 11.64. Found: C, 39.91; H, 4.20; Br, 44.25; N, 11.64.

*4,7-dibromo-2-phenyl-2H-benzo[d]1,2,3-triazole* (**m1b**): Bromination of benzotriazole **3b** (1 g, 5.13 mmol) according to the procedure described by Höger [34] afforded a pale brown solid identified as **m1b** (1.67 g, 92%). m.p.: 131–132 °C. ^1^H-NMR (CDCl_3_, ppm) δ: 8.40–8.38 (m, 2H, *o*-N-Ph), 7.64–7.54 (m 3H, *m*, *p*-N-Ph), 7.51 (s, 2H, H_BTz_). ^13^C-NMR (CDCl_3_, ppm) δ: 144.2, 139.9, 130.5, 130.0, 129.6, 120.9, 110.4. IR (neat, cm^−1^): 3067.01, 1490.96, 1201.05, 954.07, 812.5, 748.49, 664.16. MS calculated for (C_12_H_7_Br_2_N_3_) M^+^ 350.90, found 351.02. Anal. calcd. for (C_12_H_7_Br_2_N_3_): C, 40.83; H, 2.00; Br, 45.27; N = 11.90. Found: C, 40.81; H, 2.02; Br, 45.25; N = 11.92.

*2-(3,5-bis(trifluoromethyl)phenyl)-4,7-dibromo-2H-benzo[d]1,2,3-triazole* (**m1c**): Bromination of benzotriazole **6c** (1.2 g, 3.62 mmol) according to the procedure described by Höger [34] afforded a brown solid identified as **m1c** (1.31 g, 74%). m.p.: 99–100 °C. ^1^H-NMR (CDCl_3_, ppm) δ: 8.93 (s, 2H, *o*-N-Ph), 8.02 (s, 1H, *p*-N-Ph), 7.56 (s, 2H, H_BTz_). ^13^C-NMR (CDCl_3_, ppm) δ: 144.9, 140.6, 133.6, 133.5, 131.5, 123.1, 121.1, 110.6. IR (neat, cm^−1^): 3088.10, 1469.88, 1271.84, 1102.41, 939.76, 875.75, 826.81, 678.47. MS calculated for (C_14_H_5_Br_2_F_6_N_3_) M^+^ 486.88, found 486.62. Anal. calcd. for (C_14_H_5_Br_2_F_6_N_3_): C, 34.39; H, 1.03; Br, 32.68; F, 23.3; N, 8.59. Found C, 34.39; H, 1.01; Br, 32.70; F, 23.32; N, 8.58.

*2-([1,1′-biphenyl]-4-yl)-4,7-dibromo-2H-benzo[d]1,2,3-triazole)* (**m1d**): Bromination of benzotriazole **3c** (0.5 g, 1.84 mmol) according to the procedure described by Höger [34] afforded a grey solid identified as **m1d** (0.59 g, 76%). m.p.: 142–144 °C. ^1^H-NMR (CDCl_3_, ppm) δ: 8.50 (d, *J* = 8.8 Hz, 2H, *o*-N-Ph), 7.74 (d, *J* = 8.8 Hz, 2H, *m*-N-Ph), 7.61 (d, *J* = 7.3 Hz, 2H, *o*-Ph), 7.56–7.53 (m, 5H, H_BTz_, *m*-Ph, *p*-Ph). ^13^C-NMR (CDCl_3_, ppm) δ: 144.5, 141.5, 139.1, 138.5, 132.1, 130.4, 128.7, 127.8, 122.5, 121.5, 110.4. IR (neat, cm^−1^): 3378.01, 1469.88, 1194.28, 1067.02, 954.07, 805.72. MS calculated for (C_18_H_11_Br_2_N_3_) M^+^ 426.93, found 426.65. Anal. calcd. for (C_18_H_11_Br_2_N_3_): C, 50.36; H, 2.60; Br, 37.24; N, 9.79. Found: C, 50.38; H, 2.58; Br, 37.25; N, 9.78.

*4,7-bis((4-bromophenyl)ethynyl)-2-phenyl-2H-benzo[d]1,2,3-triazole* (**m2b**): The reaction between benzotriazole **4** [35] (0.100 g, 0.41 mmol) and 4-iodo-1-bromobenzene (0.232 g, 0.82 mmol) gave a yellow solid that was identified as **m2b** (0.157 g, 71%). m.p.: 166–168 °C. ^1^H-NMR (CDCl_3_, ppm) δ: 8.53 (d, 2H, *J* = 8.8 Hz, *o*-N-Ph), 7.64–7.55 (m, 13H). ^13^C-NMR (CDCl_3_, ppm) δ: 145.0, 140.1, 133.3, 131.8, 130.7, 129.5, 129.4, 123.2, 121.8, 121.1, 114.1, 95.7, 86.3. IR (neat, cm^−1^): 3067.01, 2310.99, 1476.66, 1292.92, 1059.49, 1003.01, 805.72, 748.49, 656.63. MS calculated for (C_28_H_15_Br_2_N_3_) M^+^ 550.96, found 550.22. Anal. calcd. for (C_28_H_15_Br_2_N_3_): C, 60.79; H, 2.73; Br, 28.88; N, 7.60. Found: C, 60.7; H, 2.74; Br, 28.92; N, 7.56.

*2-(3,5-bis(trifluoromethyl)phenyl)-4,7-bis((4-bromophenyl)ethynyl)-2H-benzo[d]1,2,3-triazole* (**m2c**): The reaction between benzotriazole **4 [35]** (0. 100 g, 0.26 mmol) and 4-iodo-1-bromobenzene (0.148 g, 0.52 mmol) gave a yellow solid that was identified as **m2c** (0.101 g, 56%) m.p.: 193–194 °C. ^1^H-NMR (CDCl_3_, ppm) δ: 8.93 (s, 2H, *o*-N-Ph), 8.00–7.98 (m, 5H, *p*-N-Ph and *m*-Ph), 7.54–7.52 (m, 6H, H_BTz_ and o-Ph). ^13^C-NMR (CDCl_3_, ppm) δ: 145.5, 140.8, 133.1, 131.9, 131.8, 128.4, 123.6, 121.5, 121.1, 120.6, 118.6, 114.4, 96.4, 85.8. IR (neat, cm^−1^): 3073.70, 2925.45, 1462.35, 1363.71, 1265.06, 1161.72, 989.46, 904.36, 798.19. MS calculated for (C_30_H_13_Br_2_F_6_N_3_) M^+^ 686.94, found 686.52. Anal. calcd. for (C_30_H_13_Br_2_F_6_N_3_): C, 52.29; H, 1.90; Br, 23.19; F, 16.54; N, 6.10. Found: C, 52.28; H, 1.91; Br, 23.17; F, 16.56; N, 6.08.

Polymerization procedure: Monomers (**m1–m2**) (0.3 mmol), 9,9-dihexylfluorene-2,7-diboronic acid bis(1,3-propanediol)ester (**m3**) (0.3 mmol, 150.69 mg), Pd(PPh_3_)_4_ (0.005 mmol, 5.8 mg), 1.5M K_2_CO_3_ in water (3 mmol, 2 mL), toluene (4 mL), and Aliquat (1 mL) were charged under argon to a microwave vessel. The vessel was closed and irradiated at 135 degrees for 22 min. The final mixture was cooled to room temperature and the concentrated resultant co-oligomers were precipitated in 200 mL of methanol and filtered off.

*Poly-4-(9,9-dihexyl-9H-fluoren-2-yl)-7-(9,9-dihexyl-9H-fluoren-3-yl)-2-hexyl-2H-benzo[d]1,2,3-triazole* (**P1a**): From 4,7-dibromo-2-hexyl-2*H*-benzo[*d*]1,2,3-triazole (**m1a**) (107.7 mg). After filtration the co-oligomer was obtained as a pale yellow solid (127 mg,79%). ^1^H-NMR (CDCl_3_, ppm) δ: 8.22–8.16 (brs, 2H, *o*-Ph_fluorene_), 8.15–8.09 (brs, 2H, *m*-Ph_fluorene_), 7.96–7.91 (brs, 2H, *m*-Ph_fluorene_), 7.82–7.74 (brs, 2H, H_BTz_), 4.89–4.85 (t, 2H, N-CH_2_), 2.29–2.20 (t, 2H, –CH_2_), 2.18–2.11 (t, 2H, –CH_2_), 1.52–1.47 (t, 2H, –CH_2_), 1.46–1.36 (t, 6H, 3 × –CH_2_), 1.21–1.06 (t, 12H, 6 × –CH_2_), 0.99–0.85 (m, 6H, 3 × –CH_2_), 0.81–0.74 (t, 9H, –CH_3_ × 3). IR (neat, cm^−1^): 2960.84, 2911.14, 2847.89, 2367.47, 2317.77, 1455.57, 1271.84, 819.27, 748.49.

*Poly-4-(9,9-dihexyl-9H-fluoren-2-yl)-7-(9,9-dihexyl-9H-fluoren-3-yl)-2-phenyl-2H-benzo[d]1,2,3-triazole* (**P1b**): From 4,7-dibromo-2-phenyl-2*H*-benzo[*d*]1,2,3-triazole (105.27 mg) (**m1b**). After filtration the co-oligomer was obtained as a yellow solid (61 mg, 39%) ^1^H-NMR (CDCl_3_, ppm) δ: 8.58–8.50 (brs, 2H, N-Ph), 8.33–8.27 (brs, 2H, *o*-Ph_fluorene_), 8.24–8.18 (brs, 2H, *m*-Ph_fluorene_), 8.02–7.96 (brs, 2H, *m*-Ph_fluorene_), 7.90–7.81 (brs, 2H, H_BTz_), 7.63–7.55 (brs, 2H, m-Ph), 7.54–7.47 (brs, 1H, *p*-Ph), 2.26–2.18 (brs, 4H, 2 × –CH_2_), 1.28–0.98 (brs, 16H, 8 × –CH_2_), 0.82–0.71 (brs, 6H, 2 × –CH_3_). IR (neat, cm^−1^): 2960.84, 2918.67, 2847.89, 2353.16, 2310.99, 1462.35, 1265.06, 805.72, 748.49.

Poly-2-(3,5-bis(trifluoromethyl)phenyl)-4-(9,9-dihexyl-9*H*-fluoren-2-yl)-7-(9,9-dihexyl-9*H*-fluoren-3-yl)-2*H*-benzo[d][1,2,3]triazole (**P1c**): From 2-(3,5-bis(trifluoromethyl)phenyl)-4,7-dibromo-2*H*-benzo[d]1,2,3-triazole (146.1 mg) (**m1c**). After filtration the co-oligomer was obtained as a brown solid (33 mg, 25%). ^1^H-NMR (CDCl_3_, ppm) δ: 9.03 (s, 2H, p-N-Ph), 8.32–8.28 (brs, 2H, o-Ph_fluorene_), 8.26–8.20 (br, 2H, m-Ph_fluorene_), 8.06–7.97 (brs, 3H, m-Ph_fluorene_ and p-N-Ph), 7.96–7.89 (brs, 2H, H_BTz_), 2.29–2.18 (brs, 4H, 2 × –CH_2_), 1.21–1.01 (brs, 12H, 6 × –CH_2_), 0.96–0.86 (brs, 4H, 2 × –CH_2_), 0.80–0.67 (brs, 6H, 2 × –CH_3_). IR (neat, cm^−1^): 2968.37, 2918.67, 2854.67, 2367.47, 2310.99, 1469.88, 1370.48, 1271.84, 1130.27, 897.59, 812.50.

*Poly-2-([1,1′-biphenyl]-4-yl)-4-(9,9-dihexyl-9H-fluoren-2-yl)-7-(9,9-dihexyl-9H-fluoren-3-yl)-2H-benzo[d]* [1,2,3]*triazole* (**P1d**): From 2-([1,1′-biphenyl]-4-yl)-4,7-dibromo-2*H*-benzo[*d*]1,2,3-triazole (128.07 mg) (**m1d**). After filtration the co-oligomer was obtained as a yellow solid (41 mg, 23%). ^1^H-NMR (CDCl_3_, ppm) δ: 8.64–8.50 (m, 2H, *o*-N-Ph), 8.24–8.10 (m, 4H), 7.91–7.61 (m, 8H), 7.44–7.29 (m, 3H), 2.17–2.04 (m, 8H, 4 × –CH_2_), 1.25–1.13 (m, 12H, 6 × –CH_2_), 0.88–0.84 (t, 6H, 2 × –CH_3_). IR (neat, cm^−1^): 3045.93, 2960.84, 2925.45, 2854.67, 2346.39, 2318.47, 1462.35, 1335.09, 812.50, 741.71.

Poly-4-((4-(9,9-dihexyl-9H-fluoren-2-yl)phenyl)ethynyl)-7-((4-(9,9-dihexyl-9H-fluoren-3-yl)phenyl)ethynyl)-2-phenyl-2*H*-benzo[d]1,2,3-triazole (**P2b**): From 4,7-bis((4-bromophenyl)ethynyl)-2-phenyl-2H-benzo[d]1,2,3-triazole (165.30 mg) (**m2b**). After filtration the co-oligomer was obtained as an orange solid in (75 mg, 35%). ^1^H-NMR (CDCl_3_, ppm) δ: 8.57–8.49 (d, 2H, N-Ph), 7.84–7.36 (m, 19H), 2.13–1.92 (brs, 8H, 4 × –CH_2_), 1.43–1.20 (brs, 12H, 6 × –CH_2_), 0.91–0.83 (brs, 6H, 2 × –CH_3_). IR (neat, cm^−1^): 2954.07, 2919.67, 2847.89, 2359.94, 2317.77, 2204.82, 1462.35, 1370.48, 1271.84, 812.50, 748.49.

Poly-2-(3,5-bis(trifluoromethyl)phenyl)-4-((4-(9,9-dihexyl-9H-fluoren-2-yl)phenyl)-ethynyl)-7-((4-(9,9-dihexyl-9*H*-fluoren-3-yl)phenyl)ethynyl)-2H-benzo[d]1,2,3-triazole (**P2c**): From 2-(3,5-bis(trifluoro-methyl)phenyl)-4,7-bis((4-bromophenyl)ethynyl)-2H-benzo[d]1,2,3-triazole (206.08 mg) (**m2c**). After filtration the co-oligomer was obtained as a brown solid (67 mg,26%). ^1^H-NMR (CDCl_3_, ppm) δ: 9.03 (s, 2H, o-N-Ph), 8.00 (s, 1H, p-N-Ph), 7.82–7.30 (m, 16H), 2.08–2.04 (m, 8H, 4 × –CH_2_), 1.17–1.04 (m, 12H, 6 × –CH_3_), 0.80–0.73 (t, 6H, 2 × –CH_3_). IR (neat, cm^−1^): 3094.88, 2946.57, 2911.14, 3847.89, 2211.60, 1469.88, 1370.48, 1271.84, 1165.66, 1130.27, 1003.01, 805.72, 720.63.

## 3. Results and Discussion

### 3.1. Monomer Synthesis

The corresponding monomers for 2*H*-benzo[*d*]1,2,3-triazole derivatives were synthesized by three different procedures.

**Scheme 2:**
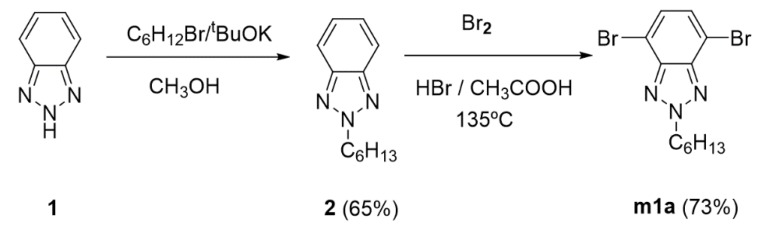
Synthetic procedure for the formation of monomer m1a.

Procedure 1: Benzotriazole **m1a** was prepared by alkylation of commercially available benzotriazole **1** with hexylbromide in the presence of ^t^BuOK and methanol to afford compound **2** (65%) [33]. Compound **2** was brominated with Br_2_ in the presence of HBr and acetic acid to give **m1a** in 73% yield (Scheme 2).

**Scheme 3:**
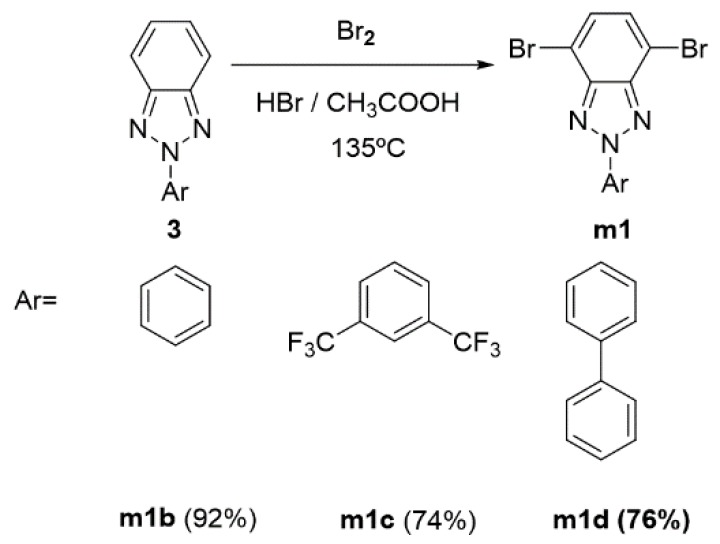
Synthetic procedure for the formation of monomers **m1b**, **m1c,** and **m1d**.

Procedure 2: Benzotriazoles **m1b**–**d** were prepared from 1-nitro-2-nitrosobenzene and the corresponding aniline, according to the synthetic method described by Höger [34], to give derivatives **3**. Bromination of **3** afforded the dibromobenzotriazoles **m1b**–**d** in excellent yields (Scheme 3).

**Scheme 4:**
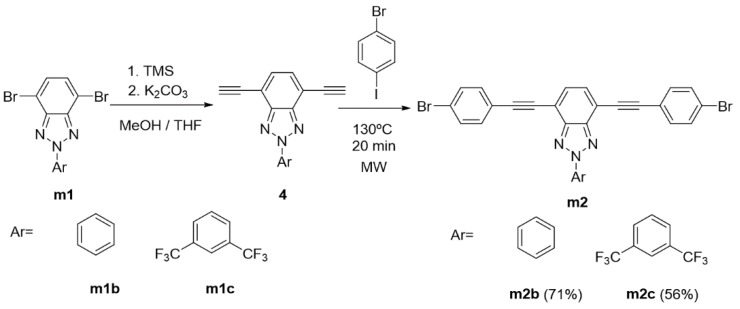
Synthetic procedure for the formation of monomers **m2b** and **m2c**.

Procedure 3: Monomers **m2b** and **m2c** were synthesized by two consecutive Sonogashira C–C cross-coupling reactions. The first reaction was between dibromobenzotriazoles **m1b** and **m1c** and ethynyltrimethylsilane (TMS) followed by deprotection with potassium carbonate [35] (Scheme 4). The second Sonogashira reaction between bis-ethynyl derivatives (**4**) and 4-iodobromobenzene using reusable Pd-EnCat TPP30, 1,5-diazabicyclo[5.4.0]undecene-5-ene (DBU), CuI and MW irradiation as the energy source afforded compounds **m2b** and **m2c** in good yields within 20 min at 130 °C (Scheme 4). This methodology has been commonly employed in our research group to prepare other benzoazole derivatives [36,37]. All the compounds gave satisfactory spectroscopic and analytical data.

### 3.2. Polymers Synthesis

Dibromo benzotriazole derivatives **m1**, **m2** (Scheme 2, Scheme 3 and Scheme 4), and 9,9-dihexylfluorene-2,7-diboronic acid bis(1,3-propanediol) ester (**m3**) reacted in presence of tetrakis(triphenylphosphine)palladium catalyst following Suzuki reaction under microwave conditions (Scheme 1) [38,39]. All compounds, designed as **P1** or **P2**, were soluble in common organic solvents such as tetrahydrofuran (THF), chloroform and dichloromethane (>1 mg/mL). Their structures were confirmed by ^1^H-NMR and IR spectroscopy, while molecular weights of each batch were determined by SEC-LS with polystyrene calibration [40], and collected in Table 1. **P1a**–**c** exhibit longer molecular lengths (n > 7 units) and thus showing a good correspondence with polymeric materials. However, **P1d** and **P2b**,**c** display shorter molecular lengths (n < 7 units) corresponding to oligomers; for instance, note that **P1b**,**c** present higher molecular weights than the corresponding **P2b**,**c.** This fact could be explained through the different reactivity of these monomers in the oxidative step, due to the larger distance of the bromide to the benzotriazole core [40,41], as the ratio of single and double alternated bonds (N#) in **P2** is double when compared to corresponding **P1**.

### 3.3. Photophysical Characterizacion

#### 3.3.1. Optical Spectroscopy Study

**Figure 1:**
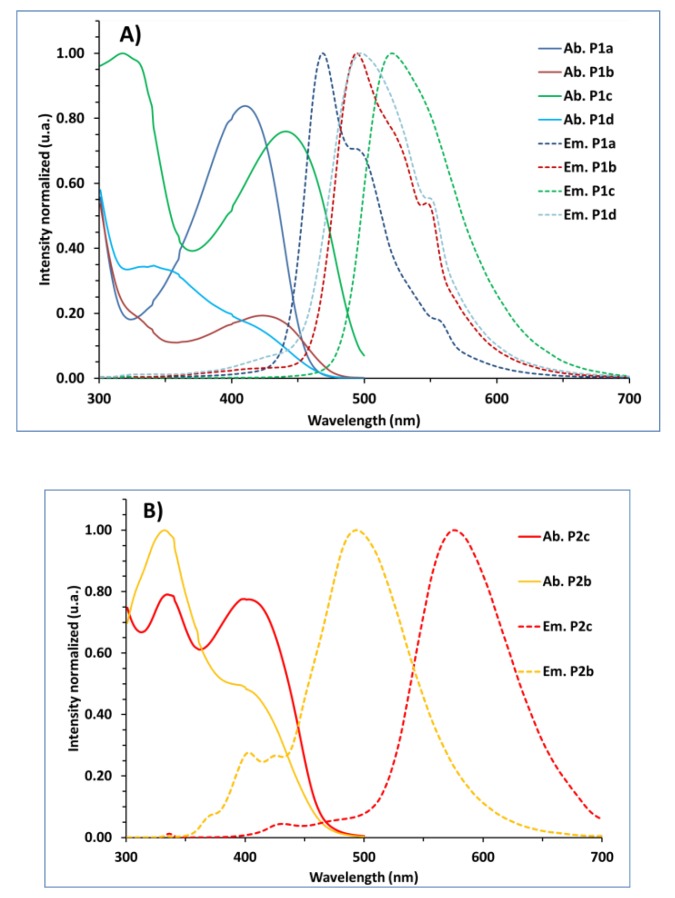
Normalized absorption (solid line) and emission (dashed line) spectra of **P1** and **P2** series at 298 K in chloroform, for (**A**): **P1a**–**d** and (**B**): **P2b**,**c**. All photoluminescence spectra were measured at maximum absorption peak of the compounds.

**Figure 2:**
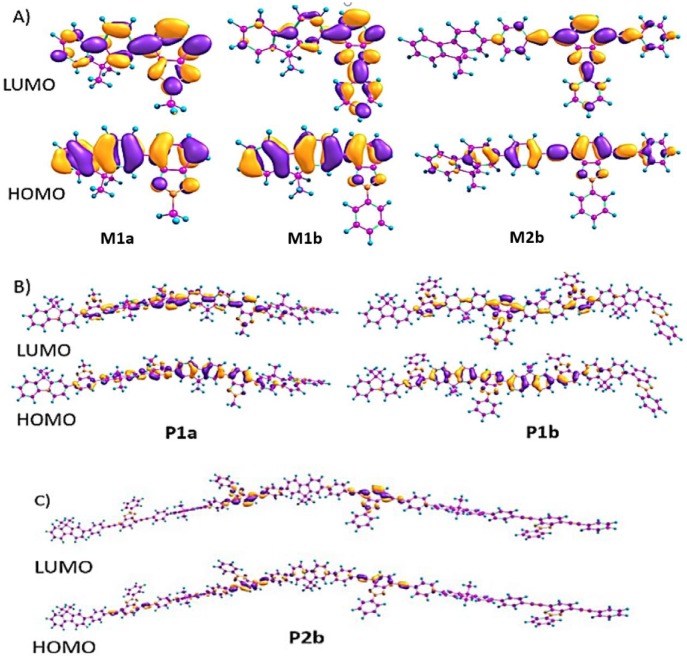
Density functional theory (DFT)-calculated frontier molecular orbitals (M06HF/6-31G** level) for **M1a**, **M1b**, and **M2b** monomers (**A**) and **P1a** and **P1b** tetramers (**B**), **P2b** tetramers (**C**). The molecular orbitals for the rest of the copolymers are given in the supporting information.

The electronic absorption and emission spectra of **P1**–**P2** were experimentally measured in CHCl_3_ solutions at a concentration of 10^−5^ M. The photoluminescence (PL) behavior was explored by exciting the molecules at their absorption maxima. In Figure 1 and Figure 2 and in Table 2, the most relevant data are collected.

The UV-visible spectra of all of the studied compounds have common features. It was observed that all D-A co-polymers showed two distinct absorption bands. The band in the low-energy region can be assigned to the intramolecular charge transfer (ICT) from the electron-donating (fluorene) unit to the benzotriazole (BTz) electron-withdrawing group. As a result of the ICT effect between the donor and acceptor moieties, all derivatives had a lower experimental optical band gap than the homopolymer poly(9,9-dihexylfluorene) (Egap = 2.92 eV) [42]. On the other hand, the high-energy absorption band corresponds to different transitions with a mixed ICT and π−π* character along the linear conjugated backbone (see Supporting Information).

TD-DFT calculations predict that the absorption band in the low-energy region is described by the transition from the ground state (S_0_) to the first excited state (S_1_), which is mainly described by a HOMO→LUMO excitation (see Tables S1–S4 in the Supporting Information). For the monomers, denoted as **M1a**–**M1d and M2b**–**M2c**, the HOMO is mostly delocalized over the π-conjugated backbone with a strong contribution from the electron-rich fluorene units, while the LUMO is mainly located on the electron-deficient BTz units (see Figure 2A). It is interesting to note that the incorporation of aryl groups on the N-2 atom of BTz results in a larger electron density localization over the BTz unit in the LUMO while slightly affecting the electron delocalization in the HOMOs (see topologies of monomers **M1a** *vs.* **M1b**). On the other hand, the insertion of alkynylphenyl groups between the donor and acceptor units results in more delocalized electron density over the alkynyl and BTz groups in the LUMOs whereas the HOMO is largely delocalized over the whole conjugated backbone (see topologies of **M1b** *vs.* **M2b**).

However, the MO topologies change significantly for longer oligomers. Whereas the LUMO is delocalized over both fluorene and BTz units in the **P1a** tetramer (see Figure 2B), the insertion of pendant aryl groups on the benzotriazole units results in a localised LUMO on the acceptor units while the HOMO is largely delocalized over the conjugated backbone core (see the MO topologies of **P1b** in Figure 2B). This situation explains the red-shift of the absorption maxima in **P1b**–**P1d** when compared to **P1a** due to the increased ICT character. On the other hand, the extension of the conjugated backbone with alkynylphenyl groups between the donor and acceptor units results in more localized HOMO and LUMO orbitals (see the MO topologies of **P2b** in Figure 2C). This situation is consistent with the observed blue-shifted absorption bands upon elongation of the conjugated backbones in **P2b**–**P2c** because of a decreased ICT character. The high electron density confinement in the HOMOs of **P2b** and **P2c** despite their more extended conjugated cores can be explained by the presence of phenyl groups. The strong aromatic character of these groups, together with their distorted structures, tends to disrupt the conjugation through the backbone. This results in larger band gaps than those found for homologous structures when only an alkynyl group connects the fluorene and BTz units (see Figures S5 and S6).

**Figure 3:**
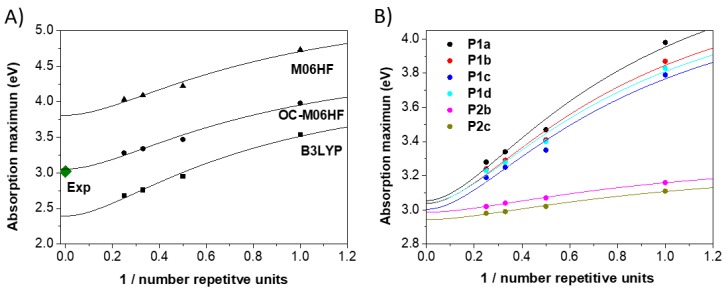
(**A**) Evolution of the S_0_→S_1_ vertical transition energy with respect to the inverse number of repeat units (1/n) at the TD-DFT level using the M06HF and B3LYP functionals for **P1a**. The OC-M06HF values with an offset correction (OC) of −0.75 are also shown. Solid lines are fitted according to the Kuhn equation. The experimental E_vert_ value, taken as the absorption band maximum of the lowest-energy transition, is shown as a green square. For the rest of the polymers, see Figure S7 in Supporting Information. (**B**) Evolution of the S_0_→S_1_ vertical transition energy with respect to the inverse number of repeat units (1/n) at the TD-DFT level using the OC-M06HF functional for **P1**–**P2**.

We next decide to investigate the optical properties at the polymer limit by using an oligomer approach, i.e., by extrapolation of the oligomer results from the monomer to the tetramer by a simple two-parameter model proposed by Kuhn [43]. As seen in Figure 3A, the B3LYP S_0_→S_1_ energies extrapolated to the polymer limit are set too low in comparison with the experimental values. The underestimation by B3LYP is ascribed to the common over-delocalization description of the wave functions in π-conjugated materials [44]. The M06HF extrapolated polymer values feature, as expected, a strong hypsochromic offset when compared to experimental values. Note that it was recently demonstrated that M06HF provides a consistent evolution with chain length for low bandgap copolymers in comparison with experimental results [45]. Since the shift is similar for all **P1**–**P2** derivatives, in this work we used the offset correction (OC) of −0.75 eV proposed earlier to compare the M06HF results with experimental data in low bandgap co-polymers [46]. It can be seen from Figure 3B that OC-M06HF extrapolated macromolecules values for the **P1**–**P2** series are in very good agreement with the experimental data.

Note that a comparison study of the optical properties of **P1**–**P2** might be feasible despite their molecular weight differences. As seen in Figure 3B, the saturation of the optical properties for **P2b**–**P2c** (with shorter chain lengths) is reached earlier as a result of the flatter curvature when compared to **P1a**–**P1d** (with higher chain lengths). This indicates that the saturation of the optical properties for systems **P2b**–**P2c** requires a lower number of repeating units when compared with systems **P1a**–**P1d** as a consequence of their backbone extension. On the other hand, for systems **P1a-P1d** similar values are found for the DFT-calculated S_0_→S_1_ energies extrapolated to the polymer limit either when using the oligomer approach from the monomer up to the tetramer, pentamer, or hexamer (see Table S5 and Figures S8–S9 in the Supporting Information). Therefore, these DFT results support that the experimental optical properties recorded for **P1a-P1d** might have reached saturation with a number of repeating units larger than 5, which is the case for all the **P1** polymers.

We also compared the experimental band gaps with those obtained theoretically by using the oligomer approach and periodic boundary conditions (PBC) method, respectively (see Figure S10 in Supporting Information). The two methods correlate well with the experimental data, thus demonstrating the ability to tune the polymer band gap as a function of the chemical structure.

**Figure 4:**
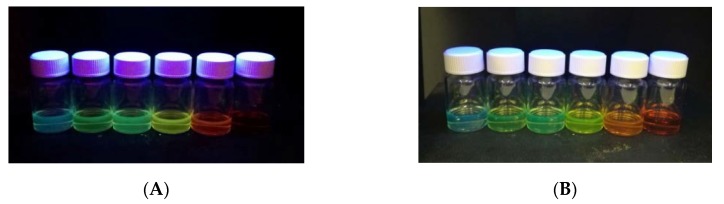
Luminescence photographs of **P1**–**P2** in CHCl_3_, upon excitation at 254 nm (**A**) and 365 nm (**B**). From left to right, the order of the compounds in the photograph is: **P1a**, **P1b**, **P1d**, **P1c**, **P2b**, and **P2c**.

The emission properties of the macromolecules were also studied. All the PL curves of the D-A chromophores contain only one resolved ICT emission band (see Figure 1). It is worth mentioning the variation in the photophysical properties as a function of the different chemical functionalization, with a bathochromic displacement observed in the following order: **P1a** < **P1b** < **P1d** < **P1c** < **P2b** < **P2c**. Thus, compounds **P2b** and **P2c**, with the more extended conjugated backbone, show maximum emission peaks at the highest wavelengths, oscillating between 500 and 600 nm. The luminescence photographs of all systems upon excitation at 254 and 365 nm are depicted in Figure 4. Compound **P1a**, with a less extended conjugated backbone, exhibits a blue color. Compounds **P1b** and **P1d** (with *N*-phenyl and *N*-biphenyl groups, respectively) show a green color. Finally, the increased ICT character upon insertion of the two CF_3_ groups in the benzotriazole core (**P1c**) or within the backbone extension (**P2b**, **P2c**) induces an extra bathochromic shift. Consequently, polymer **P1c** is yellow, **P2b** is orange and **P2c** is red. These results are in line with the theoretical bandgap predicted for the tetramers, as shown in Table 2. It is also interesting to note that all of the macromolecules are highly emissive with fluorescence quantum yields surpassing 0.50.

With the aim of explaining the emission properties, we performed geometry optimization of the S_1_ state for **M1**–**M2** monomers and their emission at the TD-DFT level (see Tables S6 and S7). Our calculations support the experimental emission spectral data with more red-shifted emission found for **P2** respect to **P1** derivatives. Note that the red-shifted transition energy found for **P2b** and **P2c** can be explained by the more extended conjugation path of the monomer in its excited state due to the presence of the alkynyl phenyl groups, which adopt a planar quinoid configuration in the S1 excited state thus allowing a better conjugation (see Table S6 and Figure S11).

Furthermore, in order to study their potential application in optoelectronic devices, emission spectra of copolymers **P1** as thin films were registered. The films were deposited by spin coating solutions of 2–5 mg/mL in chloroform on glass substrates. As shown in Figure S15, the emission spectral profiles are shifted towards lower energies but follow the same trend of those recorded in solution, indicating that fluorescence emission is not quenched by aggregation and thus supporting their potential application in solid state devices.

#### 3.3.2. Raman Spectroscopy Study

**Figure 5:**
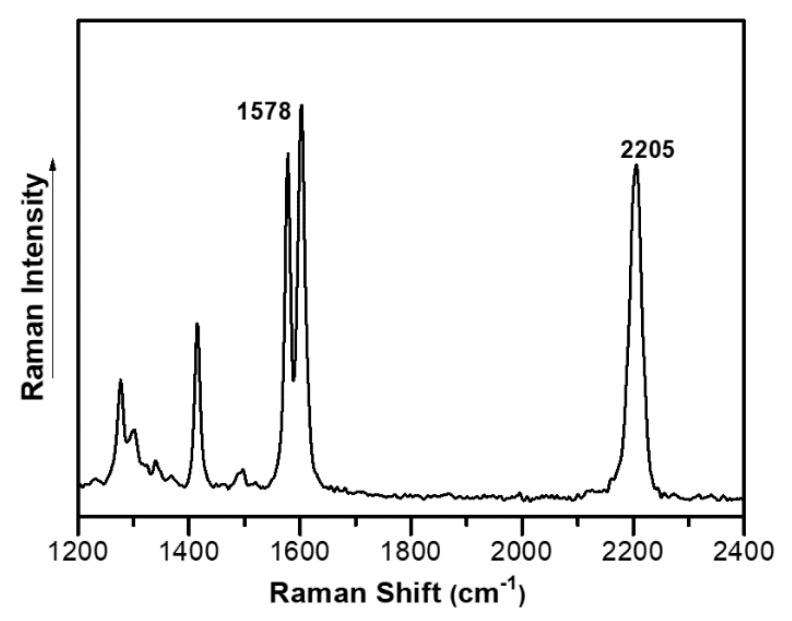
Experimental FT-Raman spectrum for **P2b**.

FT-Raman spectra were recorded to provide information about the degree of π-conjugation and intramolecular charge transfer based on the effective conjugation coordinate (ECC) theory [47,48]. This theory states that the Raman spectra of conjugated systems are dominated by a small number of vibrational modes associated with the collective C–C/C=C stretching vibrations on the conjugated backbone [49]. The Raman spectrum of compound **P2b** taken as an example (the rest of the experimental spectra are shown in the Supporting Information) is shown in Figure 5. It can be seen that the Raman spectra contain a number of clear peaks, which are assigned to particular vibrational modes with the help of density functional theory (DFT) calculations.

Specifically, we focus our discussion on the analysis of two characteristic bands collected in Table 3: The C=C/C–C stretching modes delocalized over the whole conjugated backbone involving both the fluorene and the benzotriazole groups (observed at around 1580 cm^−1^), and the C≡C stretching modes (observed at ~2200 cm^−1^) of the alkynyl groups connecting the BTz units with the donor fragments in **P2b** and **P2c**. It can be observed from the results in Table 3 that a small displacement of the C=C/C–C stretching modes located on the fluorene and benzotriazole unit is found within the **P1** polymer series. This change is associated with a moderate modulation of the electron delocalization of the π-conjugated backbones upon functionalization of the BTz units. In contrast, compounds **P2b** and **P2c**, which have a larger backbone extension, show lower wavenumber values for the aforementioned vibration, especially for **P2c**. In addition, another interesting characteristic in the **P2** series is the shift to lower frequencies of the C≡C stretching mode found in **P2c** when compared to **P2b**. This is a result of the stronger intramolecular charge transfer towards the BTz unit upon the insertion of two electron-withdrawing CF_3_ groups.

### 3.4. Fabrication and Characterization of OFETs

The evaluation of the charge transport properties of semiconducting materials is interesting to assess their potential as active materials in organic optoelectronic devices. In this section, the charge-transport properties of **P1** and **P2** were evaluated by the fabrication of top-contact/bottom-gate OFETs employing spin-coating deposition of the macromolecules solutions in chloroform on either bare Si/SiO_2_ substrates or substrates treated with an octadecyltrichlorosilane (OTS) or a hexamethyldisilazane (HMDS) self-assembled monolayer. After that, a thermal treatment called annealing was performed following by thermal gold deposition employing shadow masks in order to define the source and drain electrodes. The parameters needed to determine the electrical properties can be extracted from the I-V response plots in the saturation regime by using the assumptions of conventional transistor formalisms (Equation (1)).

The efficiency and the properties of the OFETs can be determined by the drain current in the saturation regime ((I_D_)_sat_) (Equation (1)), where W is the channel width, L the channel length, C the capacitance per unit area of the insulator layer, and V_G_ the gate voltage. The obtained parameters are field-effect mobility (µ), I_ON_/I_OFF_ ratio, and threshold voltage (V_T_) [50,51].

(I_D_)_sat_ = (W/2L)µC(V_G_ − V_T_)^2^ (1)

**Figure 6:**
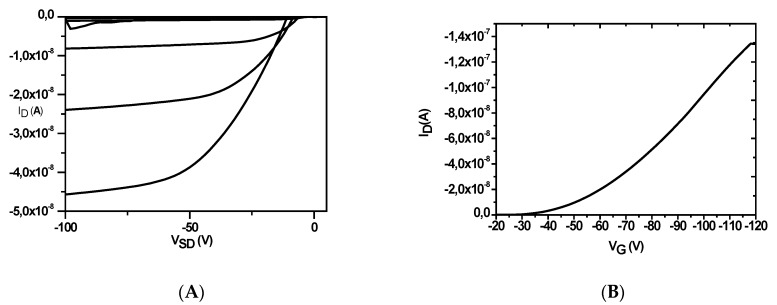
Output (**A**) and transfer (**B**) plots for **P1a**. V_G_ varies from 20 to −100 V (in 20 V steps).

The average OFET parameter data for films of **P1**–**P2** macromolecules are summarized in Table 4. These parameters were calculated using transfer plots of (I_D_)_sat_ vs. V_G_ to determine the saturation mobility, I_ON_/I_OFF_ ratio and threshold voltage for at least five devices for each sample. The parameters were calculated with V_D_ = −100 V to guarantee saturation conditions. Some representative output and transfer plots for **P1a** are shown in Figure 6.

In view of the results, it can be concluded that semiconductors **P1a**, **P1b**, **P1c**, and **P1d** show similar p-type mobilities (~10^−4^ cm^2^·V^−1^·s^−1^) after high temperature annealing treatment of the deposited films at either 180 or 240 °C. **P2b** is inactive while **P2c** shows mobilities one order of magnitude lower. This result may be ascribed to the stronger electron localization found in **P2** when compared to polymers **P1** as a result of the insertion of the alkynyl-phenyl groups between the fluorene and BTz groups. This results in deactivation of the donor and acceptor coupling within the π-conjugated core, thus hindering charge delocalization.

The active or best-performing polymer thin films were characterized by X-ray diffraction (XRD) and atomic force microscopy (AFM) techniques, which provide information about microstructural regularity and allow a good estimation of the molecular orientation with respect to the gate insulator surface. Normally the most efficient orientation between source and drain electrodes takes place when the cofacial π-conjugated molecular planes are aligned perpendicular to the dielectric substrate surface. This fact increases charge transport [52].

**Figure 7:**
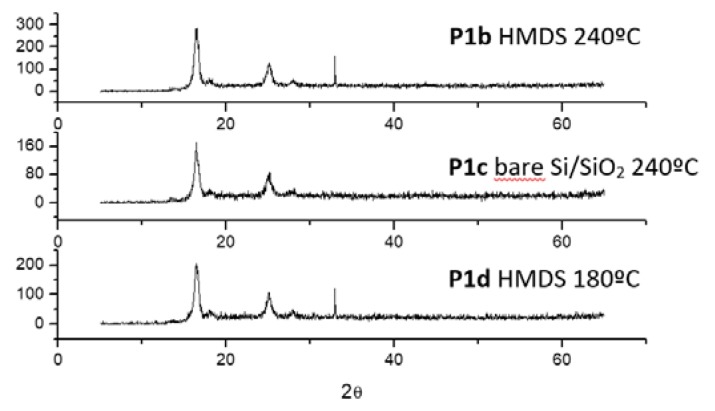
X-ray diffraction scans of vapor-deposited **P1b**, **P1c**, and **P1d** thin films. Optimal deposition conditions are indicated in the graph.

An XRD pattern of the thin films used for the OFETs is shown in Figure 7. Note that **P1b**, **P1c**, and **P1d**, in which the N-2 nitrogen atom of the benzotriazole is substituted by a phenyl derivative, show similar crystallinity patterns, with the presence of two peaks at around 17 and 25 degrees, respectively. However, substitution with phenyl groups in **P1b** polymer renders slightly most crystalline films, as the recorded XRD intensities of the peaks are larger compared to the rest of the samples. On the other hand, functionalization of the N-2 nitrogen atom of the benzotriazole with an alkyl chain, namely **P1a**, gives rise to a completely amorphous film. Nevertheless, for all of the studied polymers, the X-ray diffraction peaks are quite weak, and this indicates poorly crystalline thin film morphologies, which could be one of the reasons accounting for the recorded low electrical performances.

**Figure 8:**
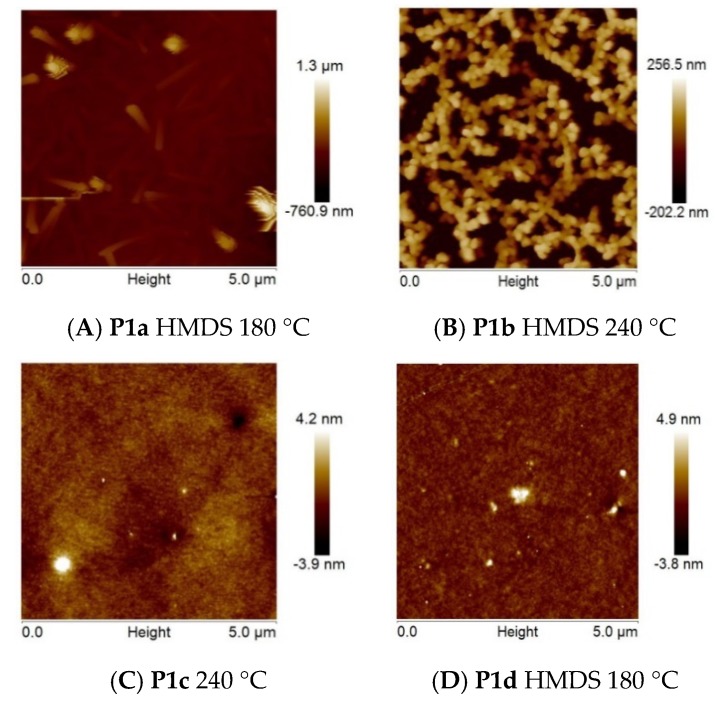
AFM images of **P1a**, **P1b**, **P1c**, and **P1d**. Image size: 5 × 5 µm.

Thin films were also characterized by tapping mode AFM. The buried dielectric-semiconductor interface, in which charge transport takes place, is the most crucial interface in a field-effect transistor. Although AFM is a superficial technique, AFM images can be used to correlate film microstructures with charge transport in the aforementioned active region. AFM images of the studied films are shown in Figure 8, showing ordered assemblies in the cases of **P1a** and **P1b**, while smaller features are recorded for the rest of the studied samples. However, as previously evidenced by X-ray studies, these aggregates differ depending on the nature of the substituent on the N-2 atom of the benzotriazole. Thus, while fibers are observed for **P1a** films, **P1b** films show well-defined round grains. Note however that the rugosity of **P1a** and **P1b** thin film samples is quite high, indicating film inhomogeneities which are detrimental for efficient charge transport. On the contrary, although less crystalline than **P1b**, more homogeneous and smoother films are observed for **P1c** and **P1d** thin film samples. Therefore, an equilibrium between film crystallinities and film homogeneities could be the reason behind the similar field-effect mobilities recorded for the **P1** series.

## 4. Conclusions

Different D-A building block of 2*H*-benzo[*d*]1,2,3-triazole and 9,9-dihexylfluorene were synthesized by a Suzuki reaction under microwave irradiation. All macromolecules obtained are highly emissive, with fluorescence quantum yields surpassing 0.50, showing color tunability from blue to red. The functionalization of the nitrogen in position 2 of the benzotriazole units or the elongation of the π-conjugated backbone through the introduction of alkynyl-phenyl groups induces a modification in the HOMO-LUMO energy and a strong modulation of the absorption and emission properties. The increased ICT character upon insertion of the two CF_3_ groups in the benzotriazole core (**P1c**) or extension of the backbone (**P2b**, **P2c**) induces an extra bathochromic shift.

DFT-calculations helped to rationalize the photophysical properties of the **P1** and **P2** derivatives. Our study demonstrates that appropriate functionalization of the benzotriazole groups in fluorene-based derivatives opens the pathway to design new D-A oligomers and polymers with color-tunable electroluminescent properties.

In addition, the studied systems were tested in OFETs in order to assess their charge transport properties for their applications in optoelectronic devices. **P1**-based polymers showed similar p-type mobilities of around 10^−4^ cm^2^·V^−1^·s^−1^. A combination of FT-Raman, AFM, and XRD studies showed that π-conjugation, planarity, packing, and film homogeneity are crucial factors that dictate the resulting electrical performances. It was also found that **P2** shows lower mobilities than **P1**. This result can be ascribed to the stronger electron localization found in oligomers **P2** when compared to **P1** as a result of the insertion of the alkynyl-phenyl groups between the fluorene and BTz groups. Furthermore, the basically amorphous polymer thin film morphologies could be one of the reasons for the moderate electrical performances shown by these systems. We hope that our results can guide the design of new benzotriazole-derived materials with potential applications in optoelectronic devices.

## Figures and Tables

**Table 1:** Molecular weights distribution of co-oligomers **P1** and **P2** obtained by SEC-LS.

Compound	*M_u_* ^a^	N# ^b^	*M_w_* ^c^	*M_n_* ^d^	*n* ^e^	PDI ^f^
**P1a**	536	6	10,771	3699	7	2.91
**P1b**	526	6	19,442	7333	14	2.65
**P1c**	662	6	9505	5001	8	1.90
**P1d**	602	6	6239	3298	5.5	1.89
**P2b**	726	12	6455	1605	2.5	4.02
**P2c**	862	12	7377	3879	4.5	1.90

^a^*. M_u_* = molecular weight unity (g/mol). ^b^. N**#**: Number of double bonds in the alternate backbone by monomer unity. ^c^*. M_w_* = weight-average molecular weight (g/mol), estimated by GPC in THF on basis polystyrene calibration. ^d^. M*_n_* = number-average molecular weight (g/mol), estimated by GPC in THF on basis polystyrene calibration. ^e^. n (number of monomer unities) = *M_n_/M_u_*. ^f^. PDI. Polydispersity = *M_w_/M_n_.*

**Table 2:** Photophysical measurement and theoretical data obtained for **P1** and **P2**.

Compound	Abs ^*a*^			Em	Φ ^*b*^	E_gap_ *^c^*	HOMO	LUMO	*E*_gap_ *^d^*
	*λ_max_*_1_	*λ_max_*_2_	*λ_onset_*	*λ_max_*		(eV)			(eV)
**P1a**	279	410	462	468	0.64	2.69	−4.85	−1.96	2.93
**P1b**	275	423	460	493	0.62	2.70	−4.94	−2.12	2.82
**P1c**	318	441	503	497	0.53	2.46	−5.20	−2.49	2.71
**P1d**	341	429	461	493	0.54	2.69	−4.94	−2.19	2.75
**P2b**	335	399	469	520	0.58	2.64	−5.07	−2.38	2.69
**P2c**	332	401	0481	574	0.51	2.58	−5.21	−2.68	2.53

*^a^.****λ*** in nm. *^b^.* **Φ** was measured in CHCl_3_ using quinine sulfate in 1M H_2_SO_4_ (**Φ** = 0.54) and 9,10-diphenylanthracene in cyclohexane (**Φ** = 0.90) as internal standards. *^c^.* The optical band gap (E_gap_) was determined from the experimental absorption onset. *^d^.* The theoretical band gap (*E*_gap_) was calculated for tetramers of **P1**–**P2** at the B3LPY/6-31g(d,p) theory level.

**Table 3:** Experimental Raman data for **P1**–**P2**.

Compound	C=C/C-C Stretching (cm^−1^)	C≡C Stretching (cm^−1^)
**P1a**	1582	-
**P1b**	1580	-
**P1c**	1579	-
**P1d**	1580	-
**P2b**	1578	2205
**P2c**	1572	2201

**Table 4:** Summary of the organic field-effect transistors (OFET) parameters for the studied polymers.

Compound	Treatment	Annealing (°C)	I_ON_/I_OFF_	V_T_ (V)	µ (cm^2^·V^−1^·s^−1^)
**P1a**	HMDS	180	3.94 × 10^3^	−47	2.23 × 10^−4^
**P1b**	HMDS	240	1.11 × 10^1^	−32	3.11 × 10^−4^
**P1c**	None	240	1.57 × 10^2^	−10	2.38 × 10^−4^
**P1d**	HMDS	180	3.36 × 10^2^	−50	2.15 × 10^−4^
**P2c**	OTS	100	6.79 × 10^2^	−85	1.79 × 10^−5^

## Supplementary Materials

The following are available online at https://www.mdpi.com/2073-4360/12/2/256/s1, Theoretical Methods; Figures S1–S5: Illustrations of the frontier molecular orbitals, Figure S6: Energy levels of the frontier molecular orbitals, Figures S7–S9: Evolution of the S_0_→S_1,_ Figure S10: Theoretical band gaps, Figure S11: DFT-B3LYP/6-31G** calculated bond-length modifications, Figure S12: DFT-calculated vertical excited state transitions at the B3LYP/6-31G** level. Figure S13: TD-DFT excitation energies. Figure S14: Theoretical FT-Raman spectra, Figure S15: Thin film emission spectra, Figures S16–S27: IR Spectra monomers and polymers, Figures S28–S45: ^1^H-NMR and ^13^C-NMR Spectra of monomers and co-oligomers. Spectra, Figures S46–S50: Experimental Raman spectra, Tables S1–S4, S7: Calculated absorption energies, Table S5: Simulated S_0_→S_1_ energies, Table S6: DFT-calculated dihedrals angles.
