# Supplementary material for: Fluorene-Based Donor-Acceptor Copolymers Containing Functionalized Benzotriazole Units: Tunable Emission and their Electrical Properties
Source: Polymers (Basel). 2020 Jan 22;12(2):256. doi: 10.3390/polym12020256 (PMC7077272; doi:10.3390/polym12020256)
Supplement: Supplementary file 1 [file polymers-12-00256-s001.pdf]

Fluorene-Based Donor-Acceptor Copolymers Containing Functionalized Benzotriazole Units: Tunable Emission and Electrical Properties

Iván Torres-Moya[a], Rebeca Vázquez Guilló[b], Sara Fernández-Palacios[c], José Ramón Carrillo[a], Ángel Díaz-Ortiz[a], J. Teodomiro López Navarrete[c], Rocío Ponce Ortiz[c], M. Carmen Ruiz Delgado[c*], Ricardo Mallavia[b*], Pilar Prieto[a*]

[a] Department of Organic Chemistry, Faculty of Chemical and Technologies Sciences, University of Castilla-La Mancha.

13071 Ciudad Real (Spain)

Email: mariapilar.prieto@uclm.es

[b] Institute of Research, Development and Innovation in Sanitary Biotechnology of Elche (IDIBE) University Miguel Hernández.

03202 Elche (Spain)

Email: r.mallavia@umh.es

[c] Department of Physical Chemistry, Faculty of Sciences, University of Málaga.

29071 Málaga (Spain)

Email: carmenrd@uma.es

1. DFT Calculations 2
2. Thin Film Solid Emission Spectra 15
3. Collection of IR spectra 16
4. Collection of NMR spectra 22
5. Collection of Raman spectra 31

1. DFT Calculations

Theoretical Methods

All calculations were carried out within the framework of the density functional theory (DFT) using the Gaussian 09 program.[1] The systems under study (**P1**, **P2**) have been modeled by using two methods: (i) the oligomer approach[2] considering oligomers from the monomer to the tetramer, and (ii) periodic boundary conditions (PBC) method. The calculations were performed by using the hybrid, generalized gradient approximation (GGA) functional B3LYP[3,4] (25% HF exchange) and the M06HF[5] (100% HF exchange) functional with in conjunction with the 6-31G** basis set.[6,7] All alkyl chains in the fluorene units were truncated to methyl groups to reduce the computational cost. To check the influence of the phenyl groups on the molecular electronic structure, two model tetramer systems analogues to **P2b** and **P2c** but without the phenyl ring connecting the alkynyl gourps with the fluorene and BTz units, named as **P2'b** and **P2'c** (see Figures S5-S6), were also computed. All geometrical parameters were allowed to vary independently, and no imaginary frequencies were observed, which ensures the finding of the global minimum energy.

The vertical electronic excitation energies were computed using the time-dependent DFT (TD-DFT) method.[8,9] In addition to the analysis of the ground-state (S_0) geometry optimizations carried out for the entire **P1** and **P2** series, optimizations of the first singlet excited state (S_1) of **M1´**-**M2´** monomers were optimized by the TD-DFT method. Molecular orbital contours were plotted using Chemcraft.[10]

References

1. M. J. Frisch et al., Gaussian 09, revision C.01: Pittsburgh, PA, 2009.
2. J. Gierschner, J. Cornil, H. J. Egelhaaf, Adv. Mater. 2007, 19, 634 173−191.
3. A. D. Becke, *J. Chem. Phys.*, 1993, 98, 5648.
4. C. Lee, W. Yang, and R. Parr, *Phys. Rev. B*, 1988, 37, 785.
5. Y. Zhao, D. G. Truhlar, J. Phys. Chem. Chem. A 2006, 110, 13126–13130.
6. P. Hariharan and J. Pople, *Theor. Chim. Acta*, 1973, 28, 213–222.
7. W. J. Hehre, R. Ditchfield, and J. A. Rople, *J. Chem. Phys.*, 1972, 56, 2257.
8. E. Runge, E. Gross, E. Physical Review Letters 1984, 52, 997–1000.
9. H. H. Heinze, A. Görling, N. Rösch, *The Journal of Chemical Physics* 2000, *113*, 2088–2099.
10. https://www.chemcraftprog.com, Chemcraft - graphical software for visualization of quantum chemistry computations.

Table S1. Calculated absorption energies (eV and nm), oscillator strengths (*f*) and major contributions for the monomers **M1'a-d** and **M2'b-c** evaluated at B3LYP/6-31G** for the ground state S_0.

Compound	E_{max} (eV)	λ_{max} (nm)	Description
**M1a**	3.54 (*f*=0.55)	350	H→L (98%)
**M1b**	3.20 (*f*=0.31)	387	H→L (98%)
**M1c**	2.92 (*f*=0.20)	425	H→L (99%)
**M1d**	3.14 (*f*=0.31)	395	H→L (98%)
**M2b**	2.65 (*f*=1.59)	467	H→L (98%)
**M2c**	2.47 (*f*=1.10)	501	H→L (99%)

Table S2. Calculated absorption energies (eV and nm), oscillator strengths (*f*) and major contributions for the monomers **M1a-d** and **M2b-c** evaluated at M06HF/6-31G** for the ground state S_0.

Compound	E_{max} (eV)	λ_{max} (nm)	Description
**M1a**	4.73 (f=0.99)	262	H→L (98%)
**M1b**	4.62 (f=0.87)	268	H→L (98%)
**M1c**	4.58 (f=0.97)	270	H→L (99%)
**M1d**	4.54 (f=0.79)	273	H→L (98%)
**M2b**	3.91 (f=2.23)	316	H→L (98%)
**M2c**	3.86 (f=2.09)	321	H→L (99%)

Table S3. Calculated absorption energies (eV and nm), oscillator strengths (*f*) and major contributions for the **P1a-d** and **P2b-c** tetramers evaluated at B3LYP/6-31G** for the ground state S_0.

Compound	E_{max} (eV)	λ_{max} (nm)	Description
**P1a**	2.68 (*f*=3.71)	462	H→L (89%)
**P1b**	2.54 (*f*=2.89)	488	H→L (88%)
**P1c**	2.38 (*f*=2.17)	520	H→L (81%)
**P1d**	2.51 (*f*=2.61)	493	H→L (87%)
**P2b**	2.41 (*f*=7.68)	514	H→L (62%)
**P2c**	2.28 (*f*=5.84)	544	H→L (54%)

Table S4. Calculated absorption energies (eV and nm), oscillator strengths (*f*) and major contributions for the **P1a-d** and **P2b-c** tetramers evaluated at M06HF/6-31G** for the ground state S_0.

Compound	E_{max} (eV)	λ_{max} (nm)	Description
**P1a**	4.03 (*f*=5.74)	307	H→L (47%)
**P1b**	3.98 (*f*=5.38)	311	H→L (45%)
**P1c**	3.94 (*f*=5.08)	314	H→L (38%)
**P1d**	3.98 (*f*=5.13)	311	H→L (43%)
**P2b**	3.77 (*f*=10.65)	328	H→L (25%)
**P2c**	3.73 (*f*=10.05)	332	H→L (21%)

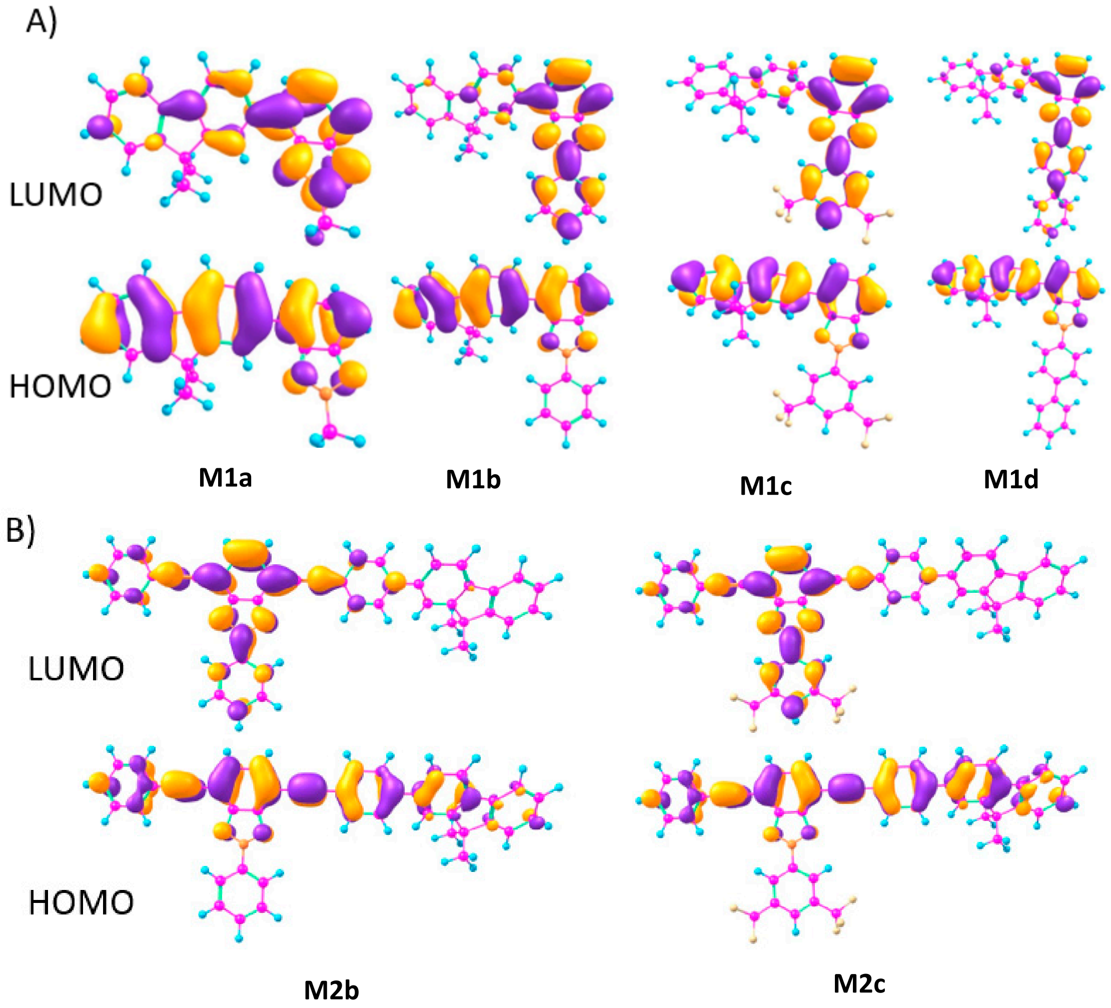

Figure S1. Illustrations of the frontier molecular orbitals for **M1a-M1d** (a) and **M2b-M2c** monomers evaluated at the B3LYP/6-31G** level.

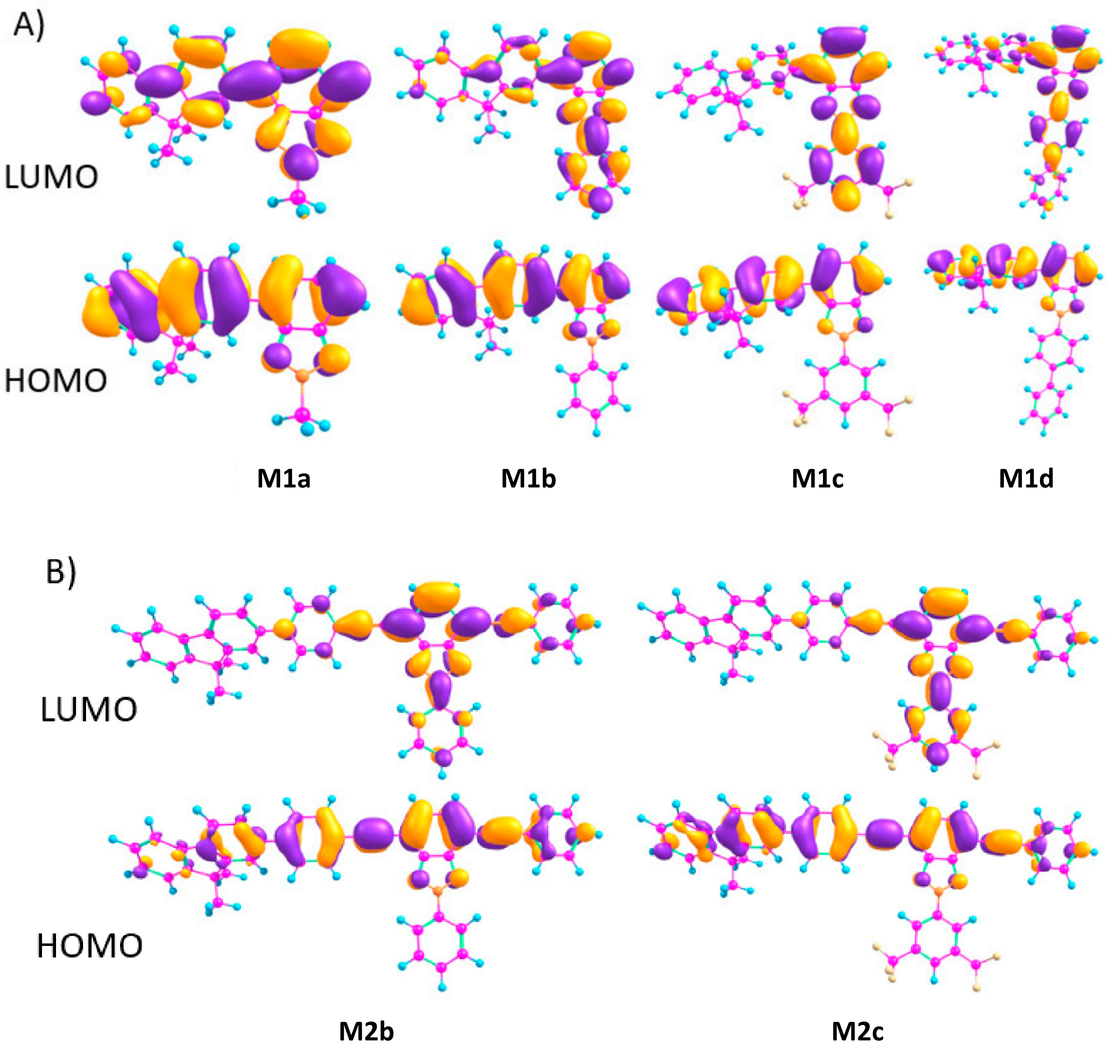

Figure S2. Illustrations of the frontier molecular orbitals for **M1a-M1d** (a) and **M2b-M2c** (b) monomers evaluated at the M06HF/6-31G** level.

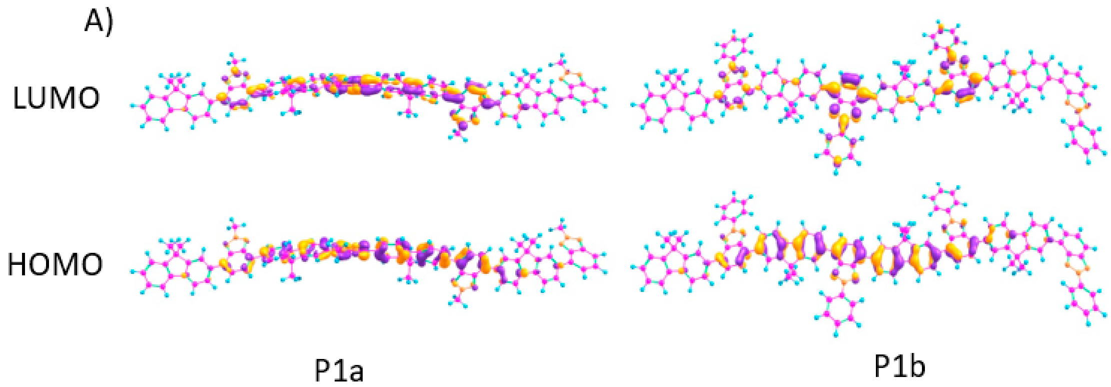

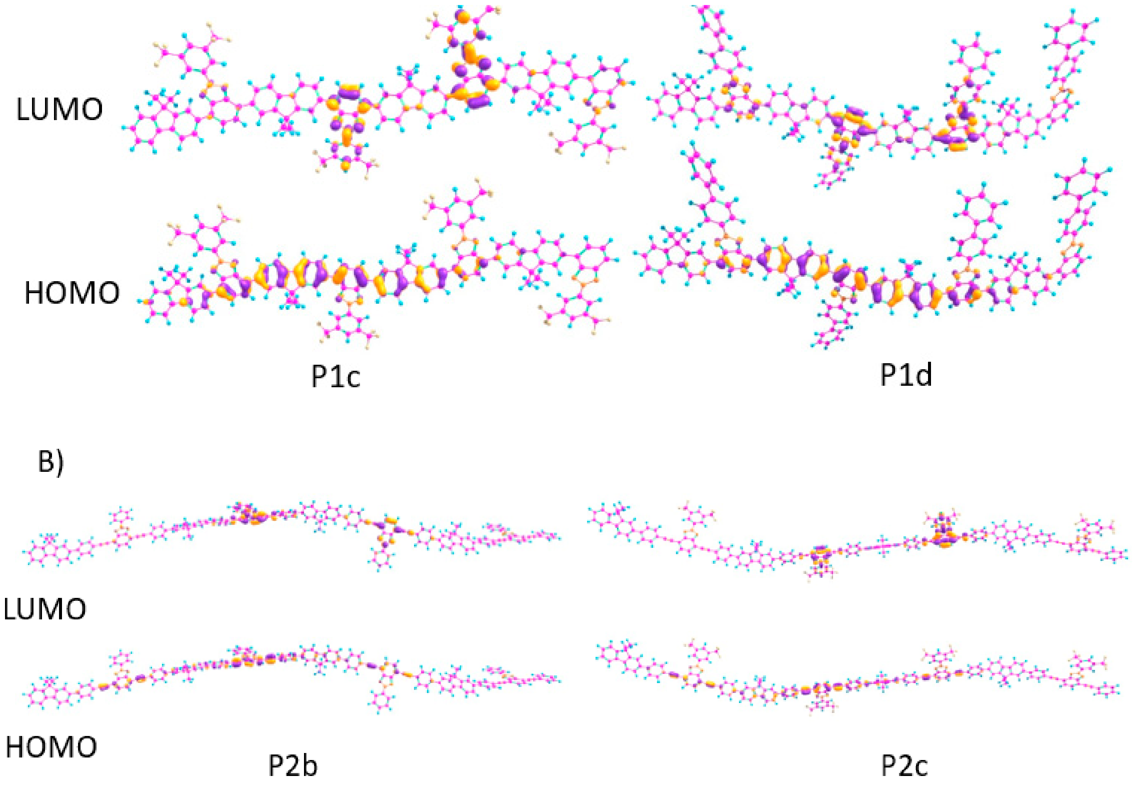

Figure S3. Illustrations of the frontier molecular orbitals for **P1a-P1d** (b) and **P2b**-**P2c** (b) tetramers evaluated at the B3LYP/6-31G** level.

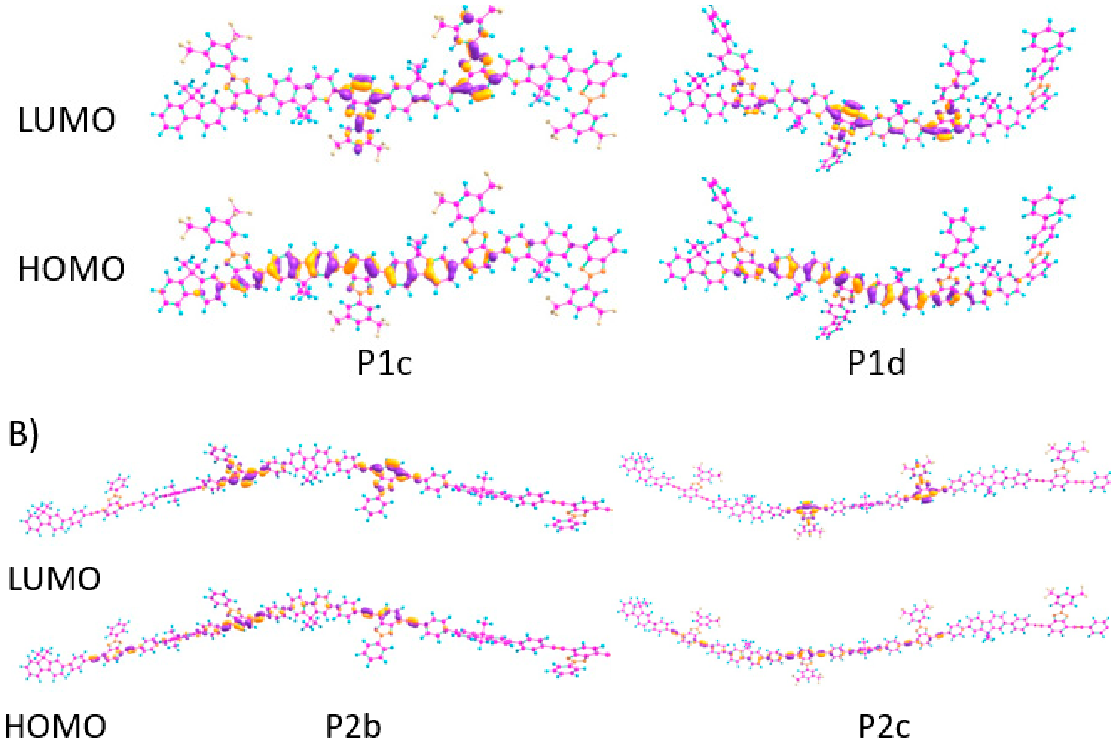

Figure S4. Illustrations of the frontier molecular orbitals for **P1a-P1d** (a) and **P2b**-**P2c** (b) tetramers evaluated at the M06HF/6-31G** level.

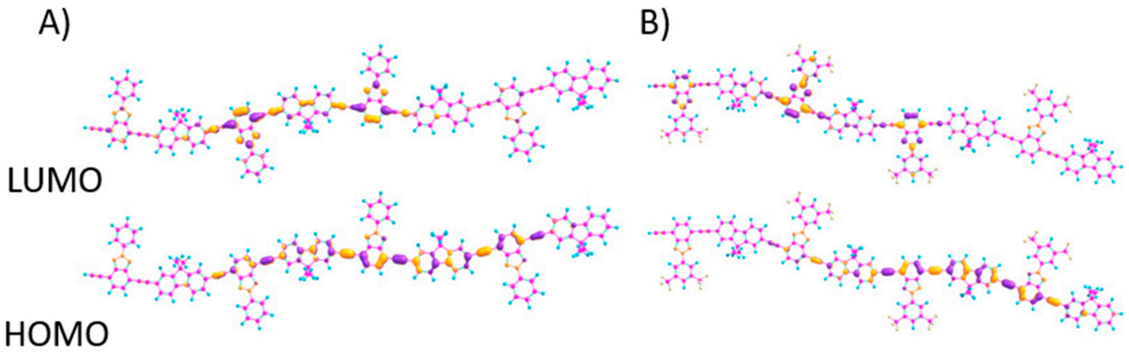

Figure S5. Illustrations of the frontier molecular orbitals (B3LYP/6-31G** level) for tetramers of homologues copolymers of **P2b** and **P2c** without the phenyl groups between the alkynyl and fluorene units, denoted as **P2b’** and **P2c’**, respectively.

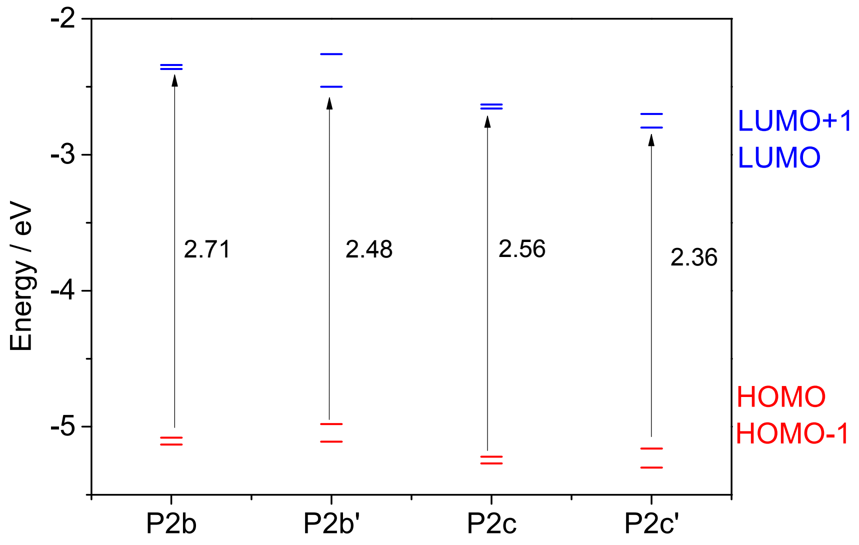

Figure S6. Energy levels of the frontier molecular orbitals (B3LYP/6-31G** level) for tetramers of **P2b** and **P2c** copolymers and their homologues copolymers without the phenyl groups between the alkynyl and fluorene units denoted as **P2’b and P2’c**.

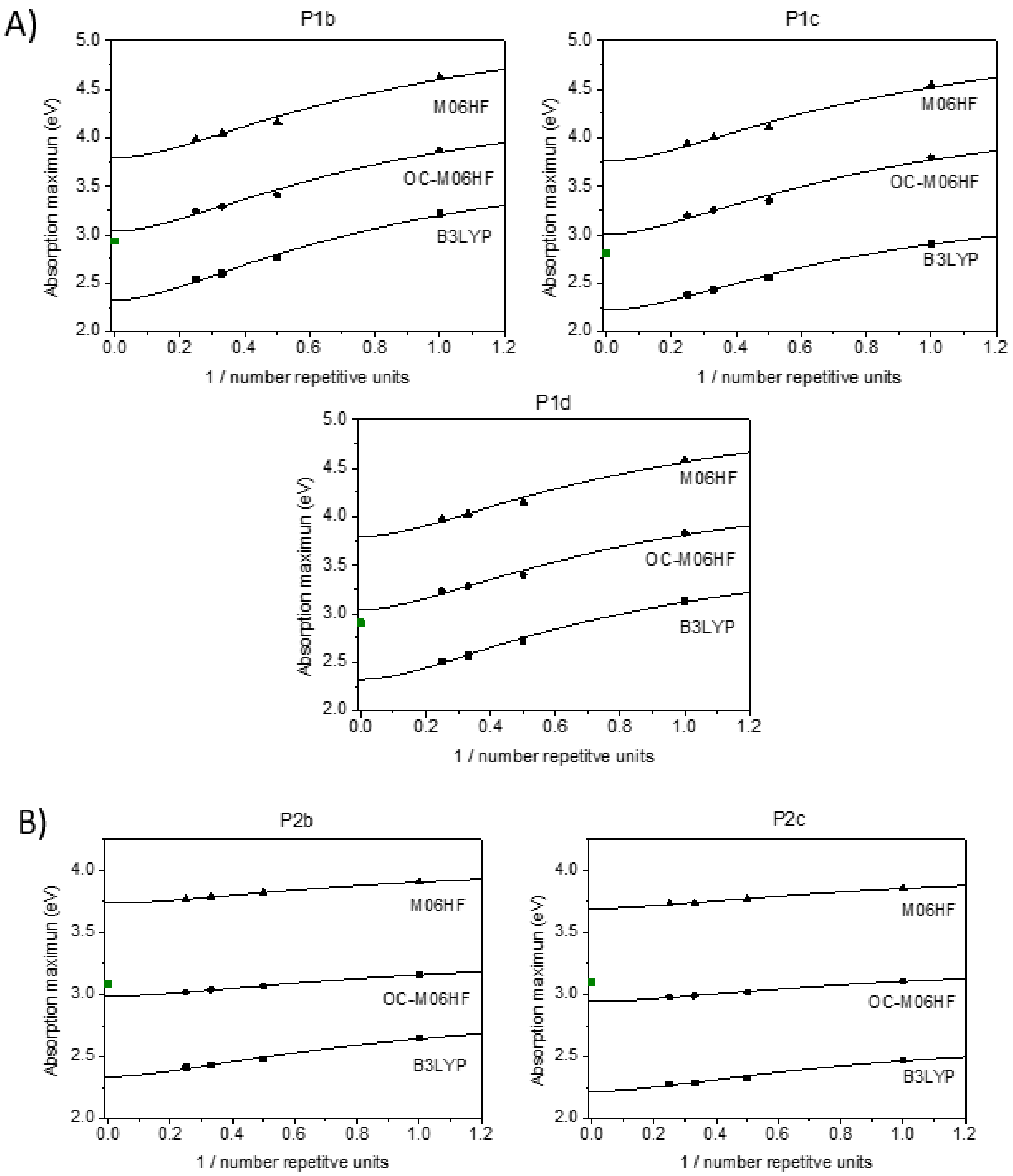

Figure S7. Evolution of the $S_0 \rightarrow S_1$ vertical transition energy with respect to the inverse number of repeat repeat units (1/n) at the TD-DFT level using the M06HF and B3LYP functionals for **P1b-P1d** copolymers. The OC-M06HF values with an offset correction (OC) of -0.75 are also shown. Solid lines are fit according to the Kuhn equation. B) Evolution of the $S_0 \rightarrow S_1$ vertical transition energy with respect to the inverse number of repeat units (1/n) at the TD-DFT level using the OC-M06HF functional for **P2b**-**P2c** copolymers. The experimental E_{vert} values, taken as the absorption band maximum of the lowest-energy transition, are shown as green squares.

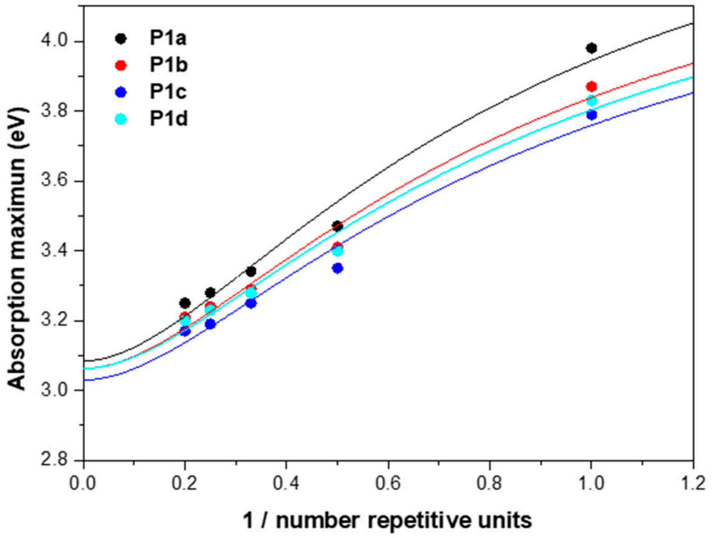

Figure S8. Evolution of the $S_0 \rightarrow S_1$ vertical transition energy with respect to the inverse number of repeat units (1/n) at the TD-DFT level using the OC-M06HF functional for copolymers **P1** by using and oligomer approach from the monomer to the pentamer.

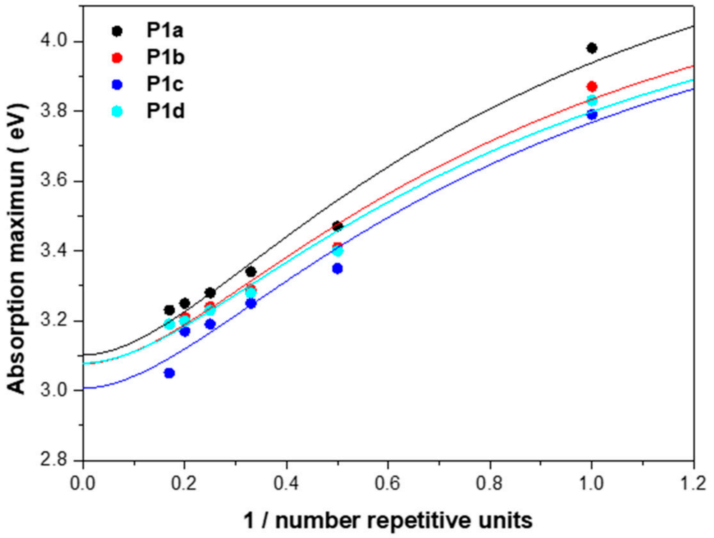

Figure S9. Evolution of the $S_0 \rightarrow S_1$ vertical transition energy with respect to the inverse number of repeat units (1/n) at the TD-DFT level using the OC-M06HF functional for copolymers **P1** by using and oligomer approach from the monomer to the hexamer.

Table S5. Simulated $S_0 \rightarrow S_1$ energies extrapolated to the polymer limit in polymers **P1a**-**P1d**, calculated at the TD-DFT level using the OC-M06HF functional, by using an oligomer approach from the monomer to the tetramer, pentamer or hexamer, respectively.

DFT-calculated $S_0 \rightarrow S_1$ energies extrapolated to the polymer limit			
**Polymer**	**Monomer to Tetramer**	**Monomer to Pentamer**	**Monomer to Hexamer**
**P1a**	3.05	3.08	3.10
**P1b**	3.03	3.06	3.07
**P1c**	3.00	3.03	3.00
**P1d**	3.04	3.06	3.07

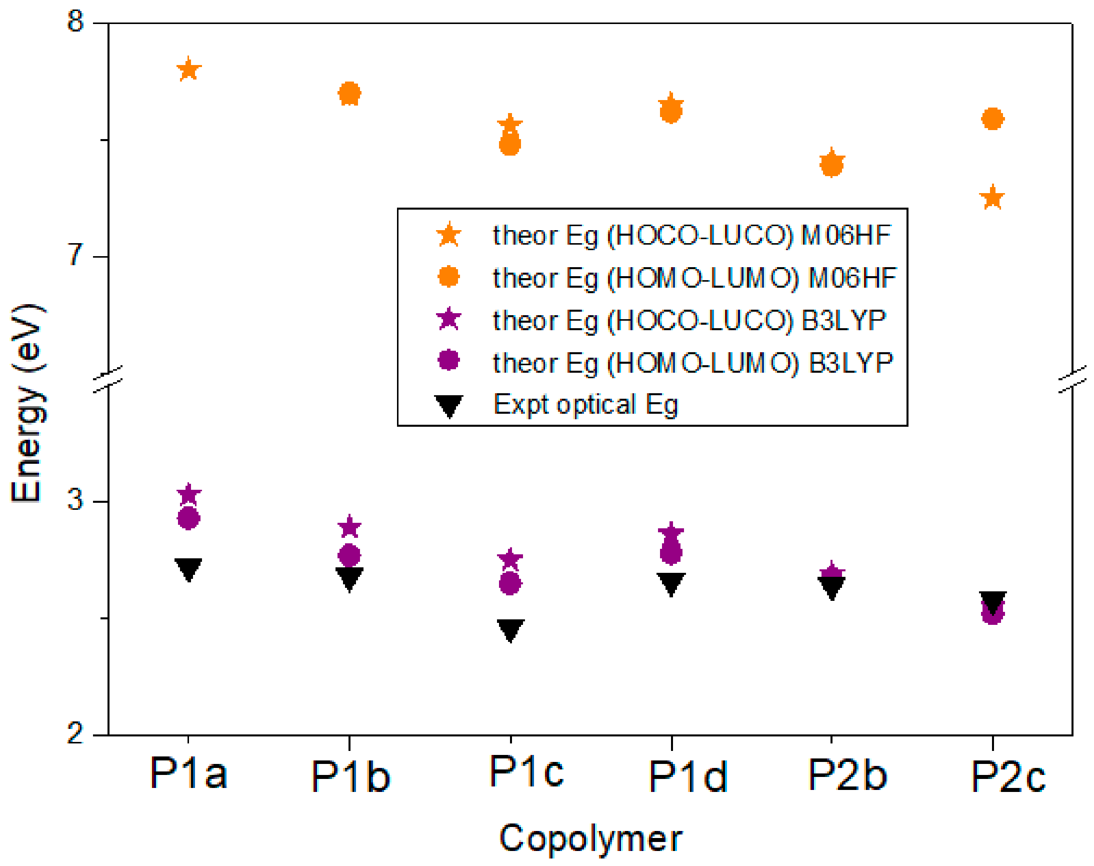

Figure S10. Theoretical band gaps for **P1b-P1d** and **P2b-P2c** copolymers, obtained as the difference between the HOMO and LUMO values and the HOCO and LUCO values. HOMO and LUMO values for the polymers were obtained from Kuhn fits using model oligomers (from n =1 to n=4). -6). For the calculation of the highest occupied crystal orbital (HOCO) and lowest unoccupied crystal orbital (LUCO) values, DFT calculations using periodic boundary conditions were used. The experimental band gap values were determined directly from absorption spectrum.

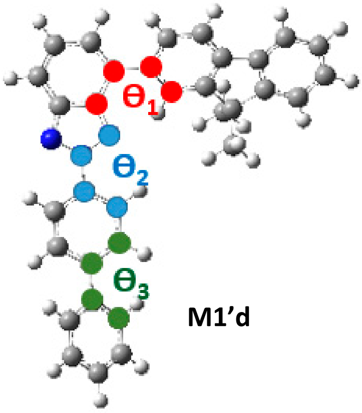

M1'd

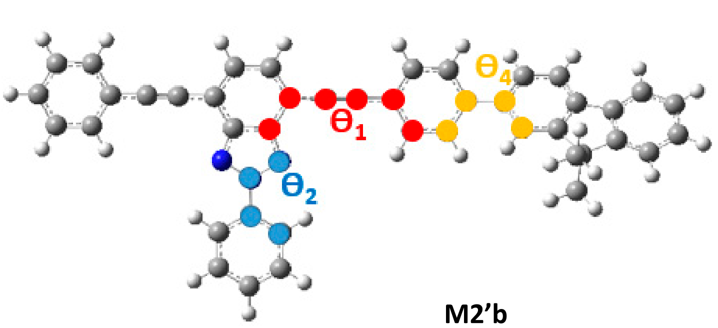

M2'b

Table S6. DFT-calculated dihedrals angles (B3LYP/6-31G** level) for the monomers **M1a-d** and **M2b-c** in the ground state S_0 and the first excited state S_1 (values shown in parenthesis).

**Compound**	**θ_1**	**θ_2**	**θ_3**	**θ_4**
**M1a**	33 (6)			
**M1b**	34 (9)	4 (1)		
**M1c**	34 (14)	2 (0)		
**M1d**	34 (8)	5 (2)	37 (31)	
**M2b**	0 (0)	0 (1)		36 (26)
**M2c**	0 (0)	0 (1)		36 (28)

Table S7. Calculated emission energies (eV and nm), oscillator strengths (*f*) and major contributions for the polymers **P1a-P1d**, **P2b** and **P2c** evaluated at B3LYP/6-31G** for the ground state S_1.

**Compound**	**E_{max} (eV)**	**λ_{max} (nm)**	**Description**
**P1a**	2.89 (*f*=0.52)	428	H→L (98%)
**P1b**	2.61 (*f*=0.33)	475	H→L (98%)
**P1c**	2.37 (*f*=0.21)	522	H→L (99%)
**P1d**	2.55 (*f*=0.34)	486	H→L (98%)
**P2b**	2.32 (*f*=1.60)	534	H→L (98%)
**P2c**	2.12 (*f*=1.00)	583	H→L (98%)

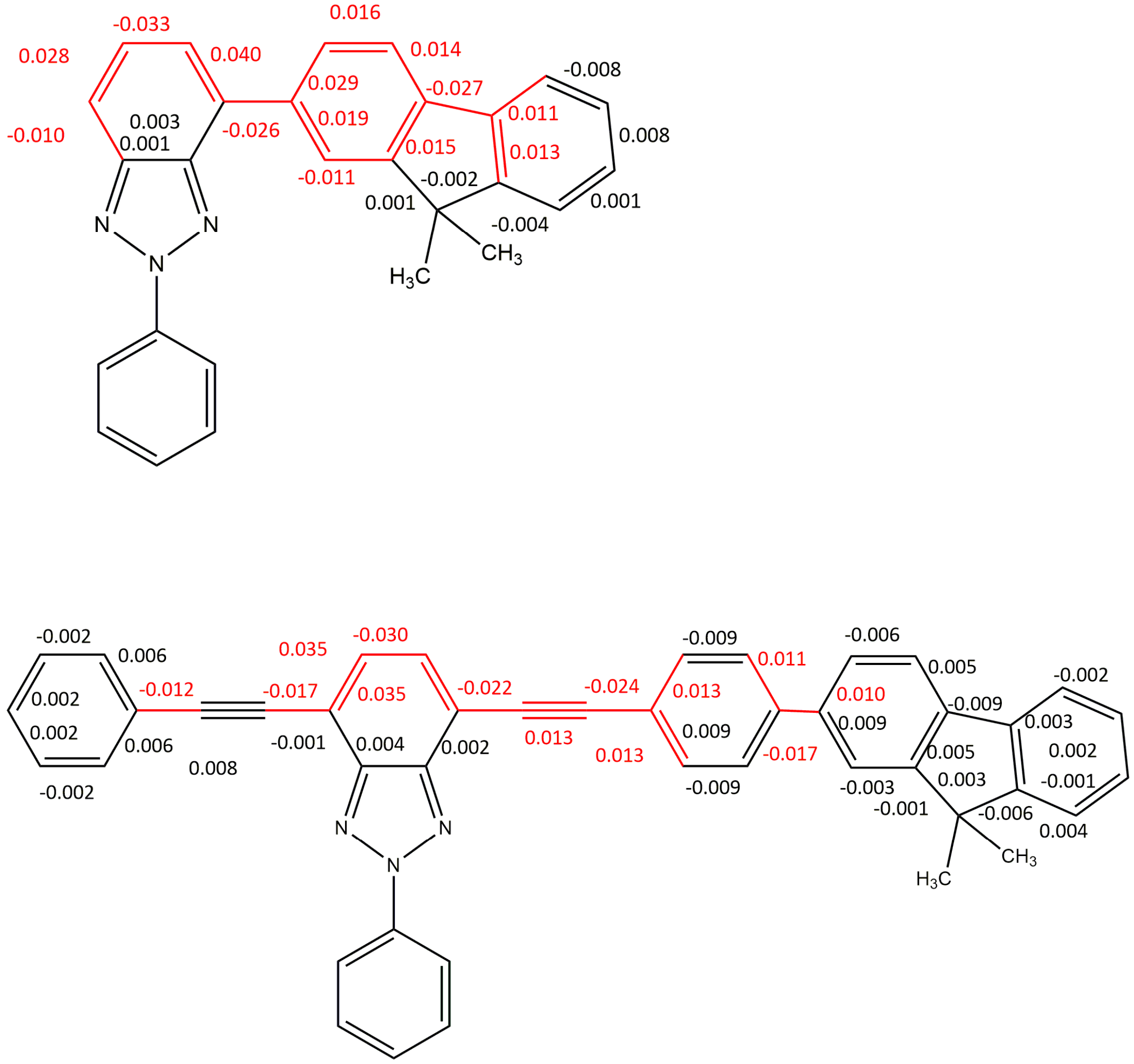

Figure S11. DFT-B3LYP/6-31G** calculated bond-length modifications (Å) for **M1b** (top) and **M2b** (bottom) molecules on going from S_1 to S_0. The bond length modifications (Δx) larger than 0.010 Å are highlighted in red.

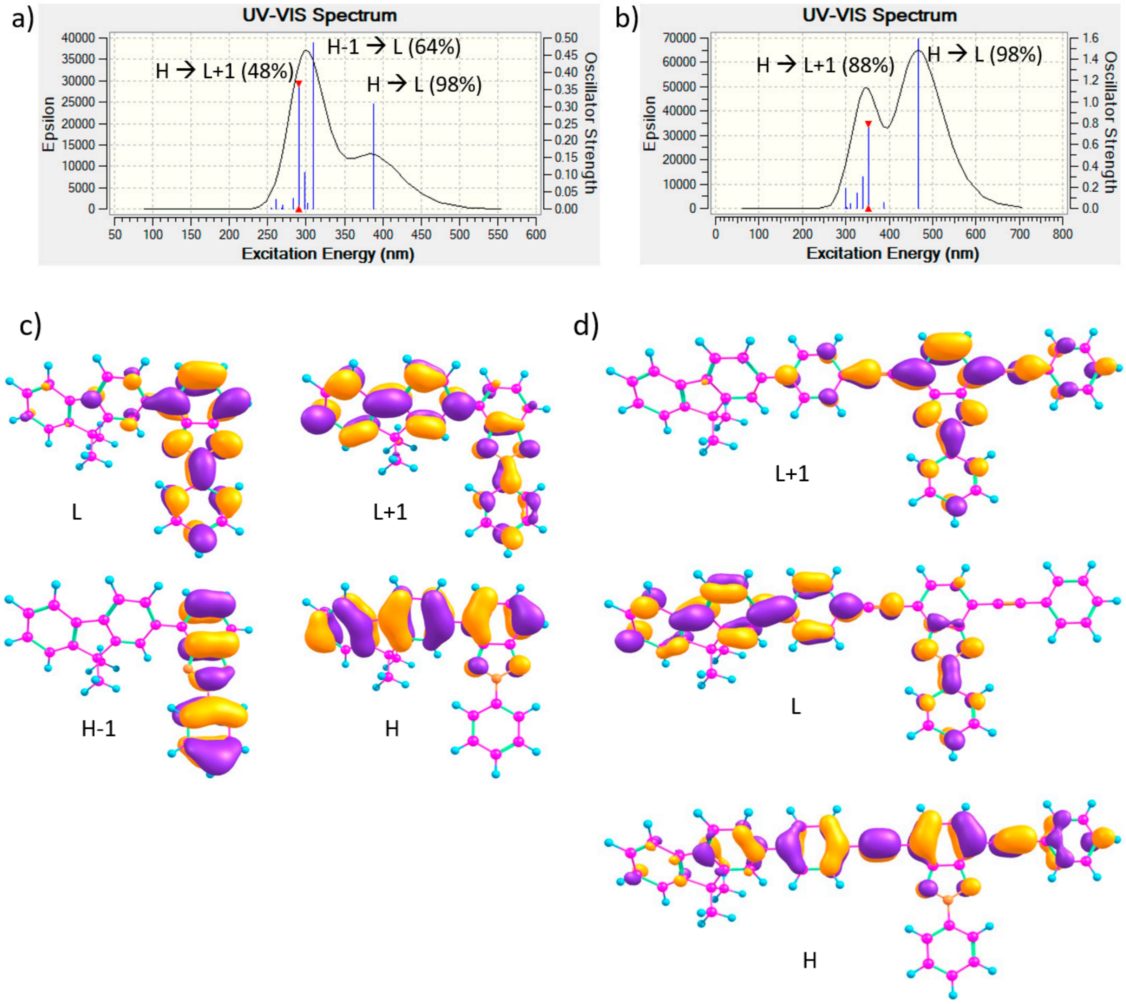

Figure S12. DFT-calculated vertical excited state transitions at the B3LYP/6-31G** level for M1b (a) and M2b (b). b) The frontier molecular orbitals topologies involved in the main electronic transitions of M1b (c) and M2b (d) are also shown.

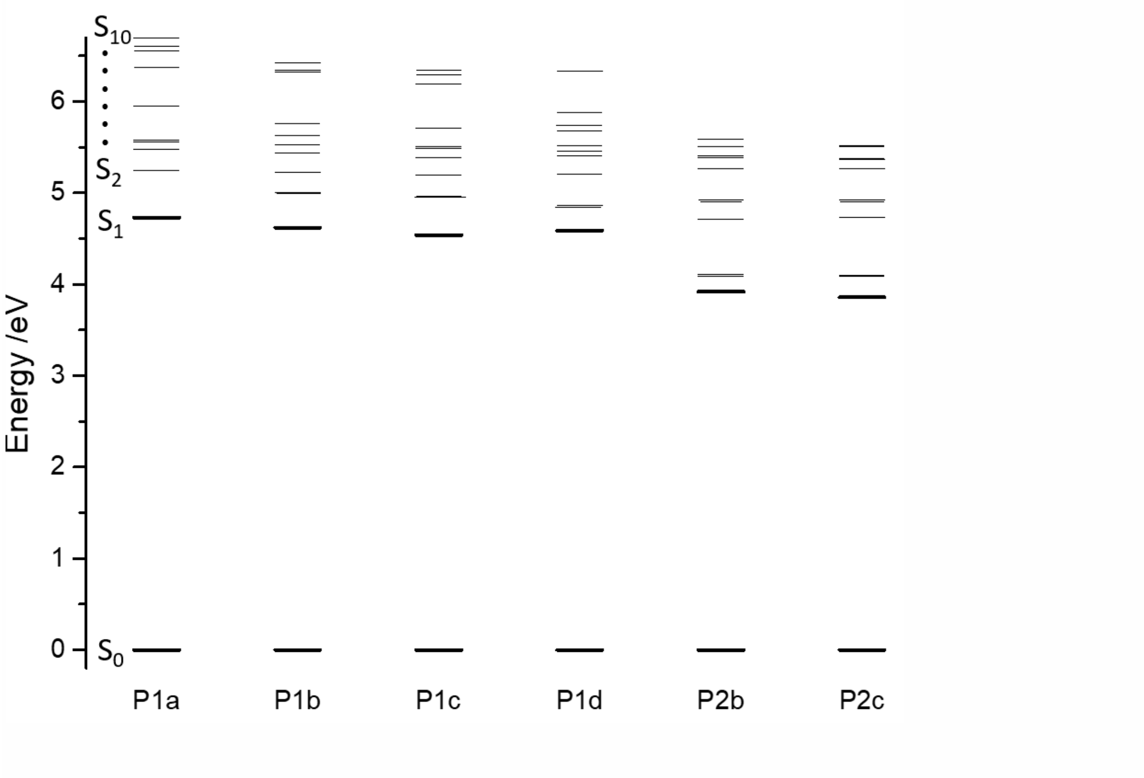

Figure S13. TD-DFT excitation energies (B3LYP/6-31G** level) for **P1** and **P2** tetramers.

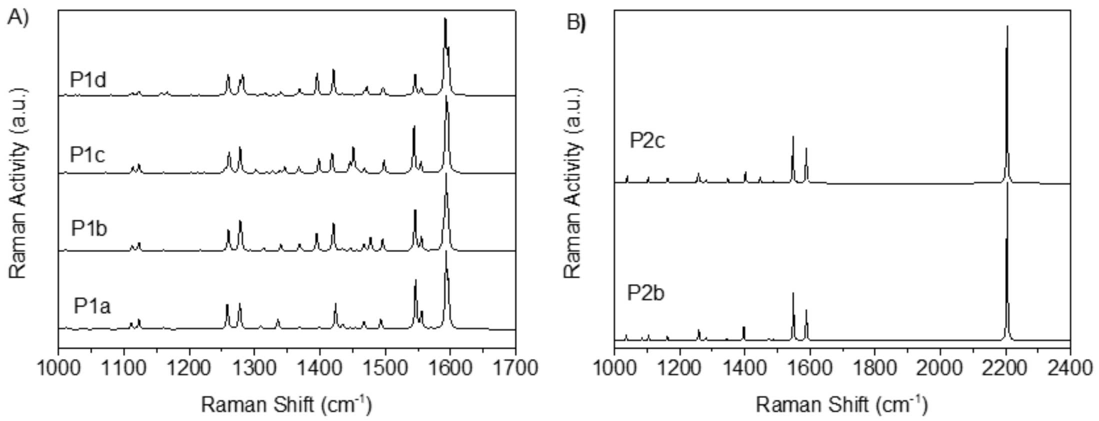

Figure S14. Theoretical FT-Raman spectra for polymers **P1a-P1d** (a) and **P2b-P2c** (b) evaluated at B3LYP/6-31G**.

2. Thin Film Solid Emission Spectra

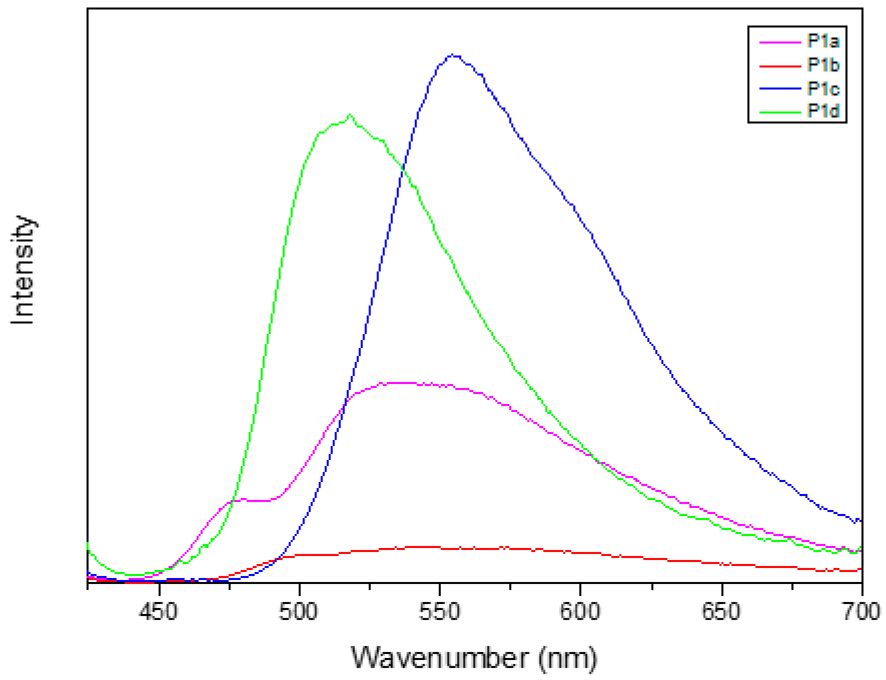

Figure S15. Thin film emission spectra of **P1** polymers recorded after excitation at their corresponding maximum absorption peaks.

3. Collection of IR Spectra

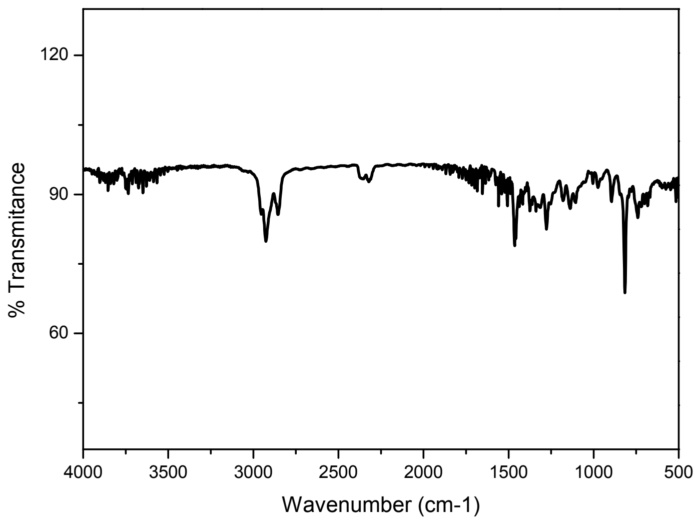

Figure S16. IR Spectra of polymer **P1a**.

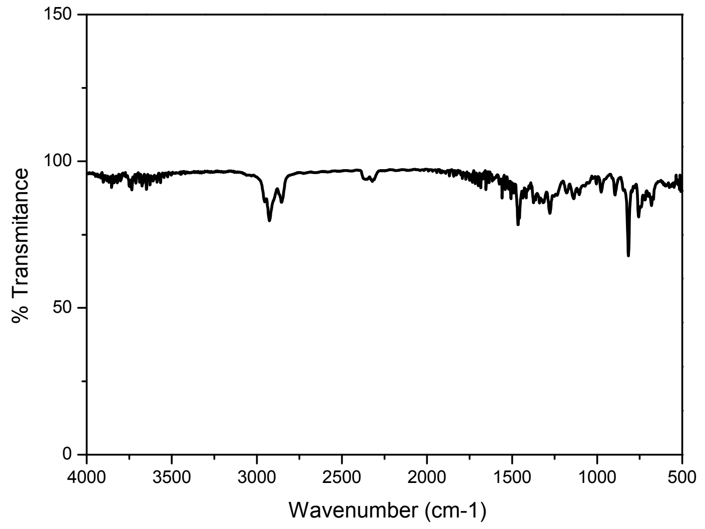

Figure S17. IR Spectra of polymer **P1b**

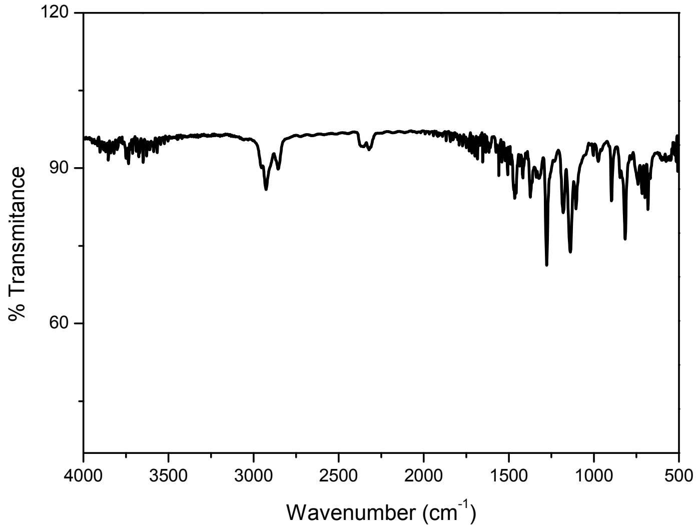

Figure S18. IR Spectra of polymer **P1c**

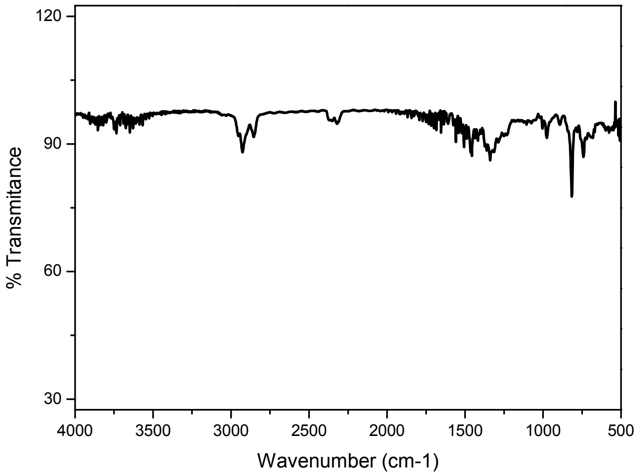

Figure S19. IR Spectra of polymer **P1d**

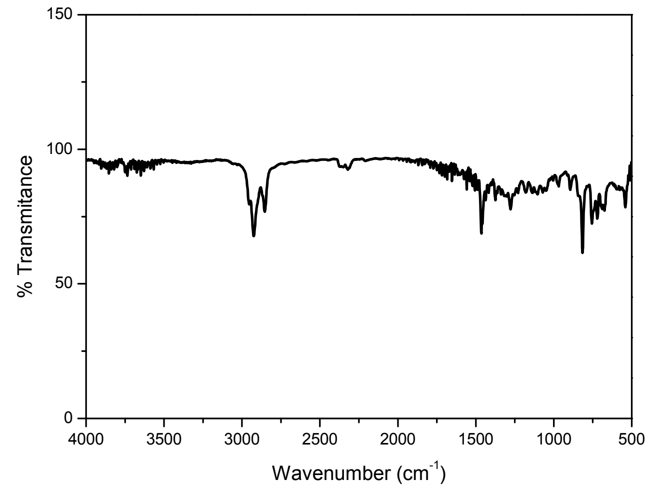

Figure S20. IR Spectra of polymer **P2b**

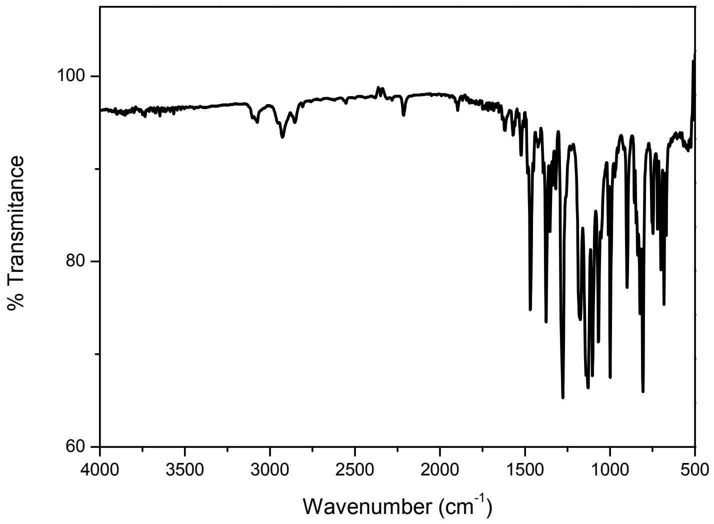

Figure S21. IR Spectra of polymer **P2c**

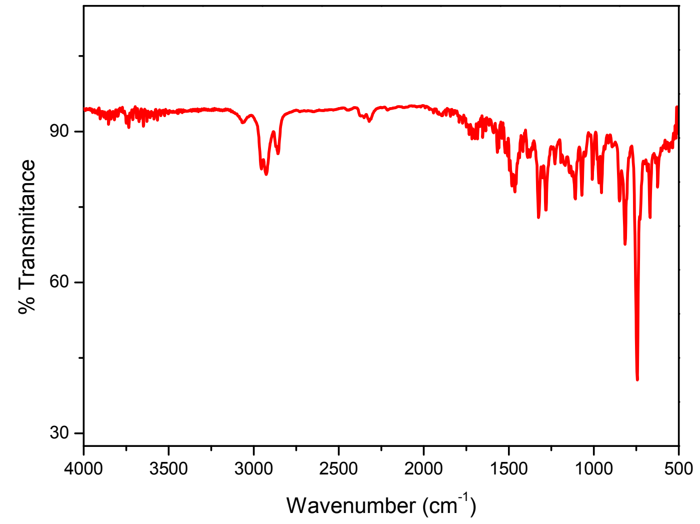

Figure S22. IR Spectra of monomer **m1a**

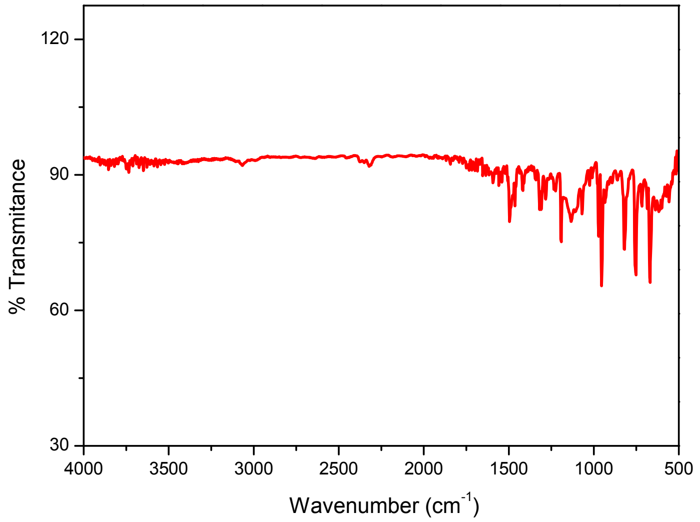

Figure S23. IR Spectra of monomer **m1b**

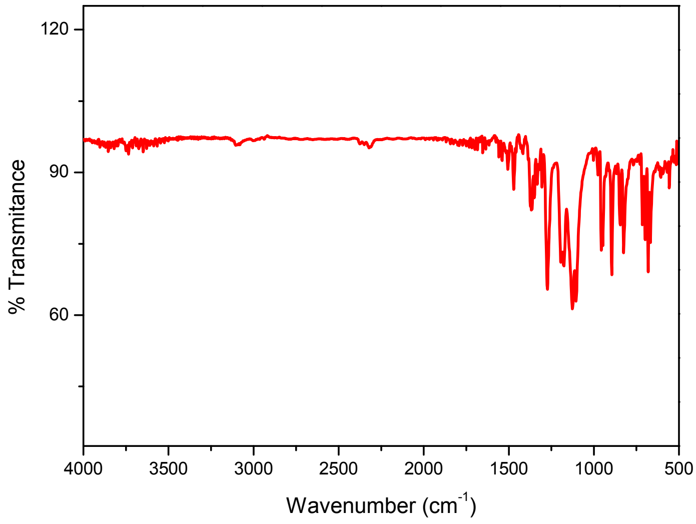

Figure S24. IR Spectra of monomer **m1c**

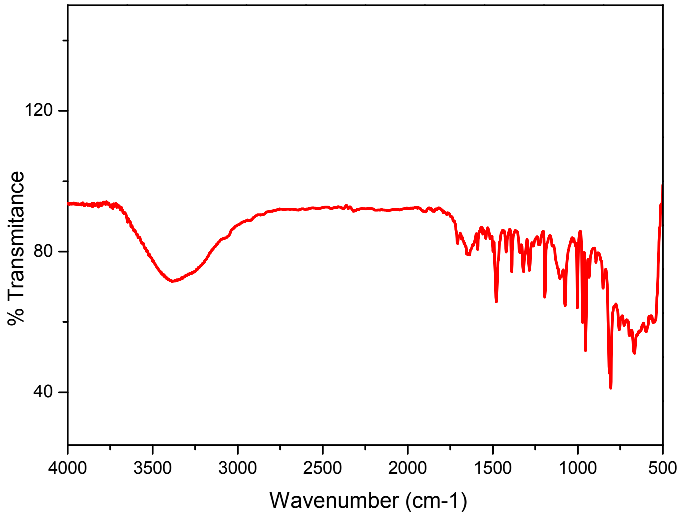

Figure S25. IR Spectra of monomer **m1d**

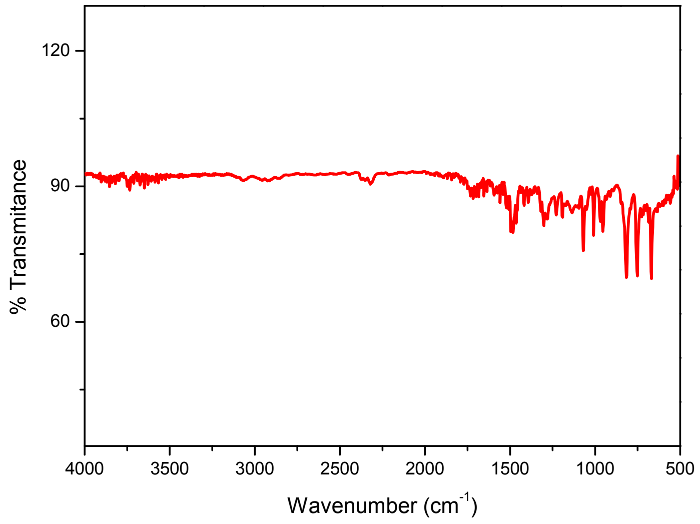

Figure S26. IR Spectra of monomer **m2b**

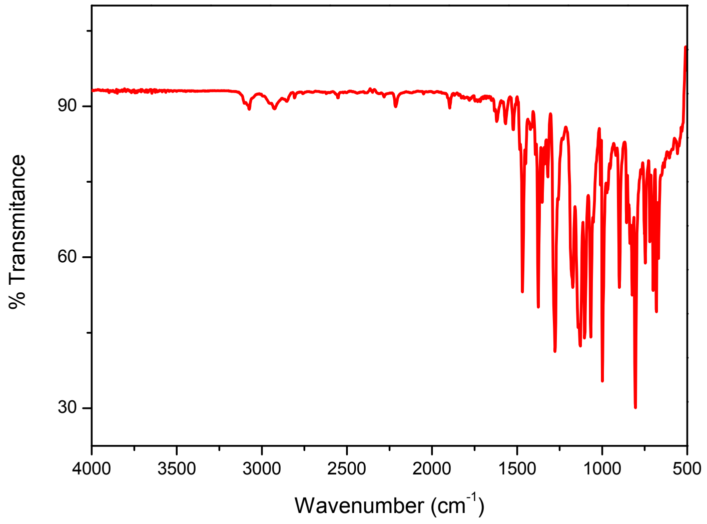

Figure S27. IR Spectra of monomer **m2c**

4. Collection of NMR spectra

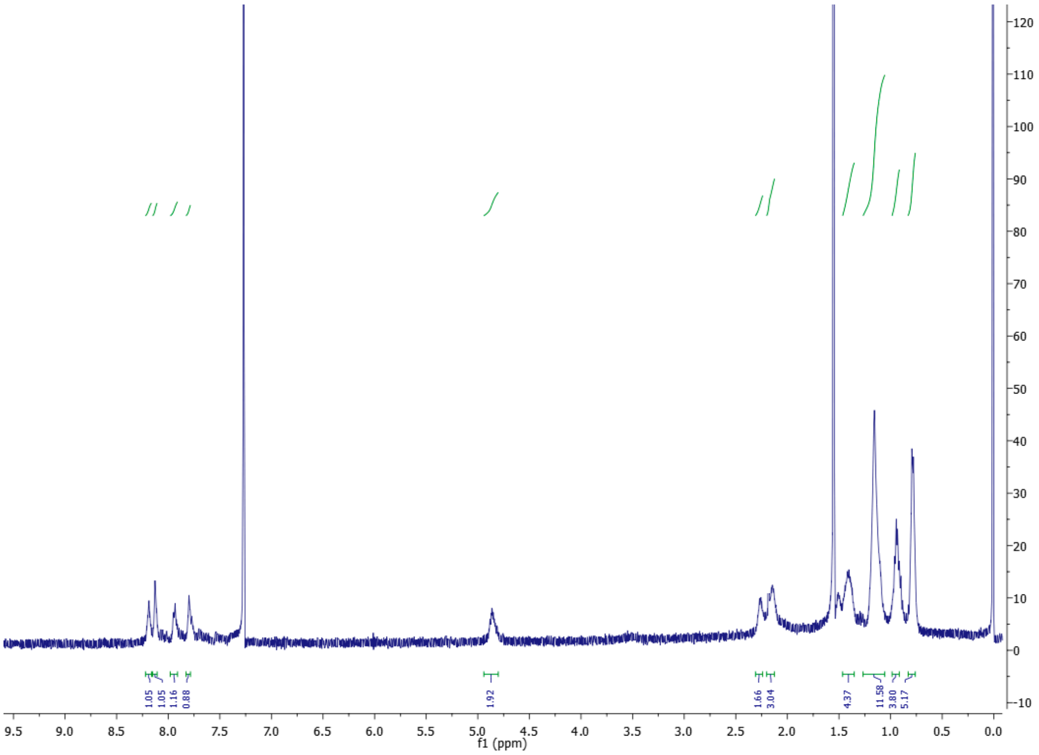

Figure S28. ^{1}H-NMR Spectra of polymer **P1a**

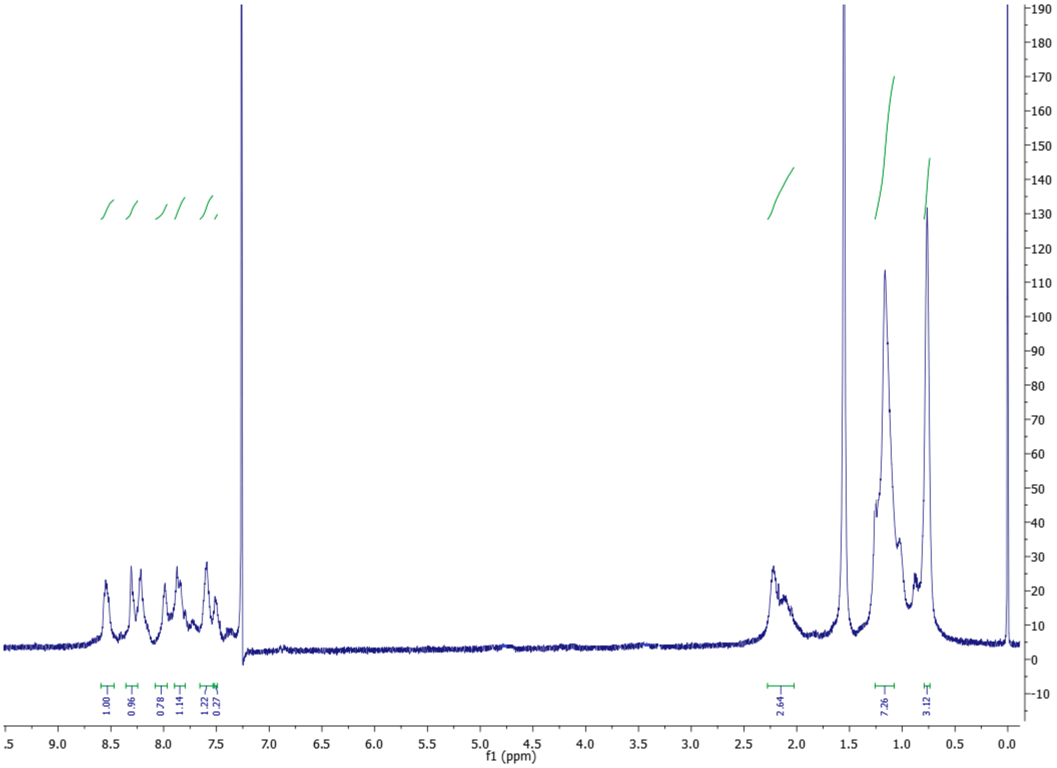

Figure S29. ^{1}H-NMR Spectra of polymer **P1b**

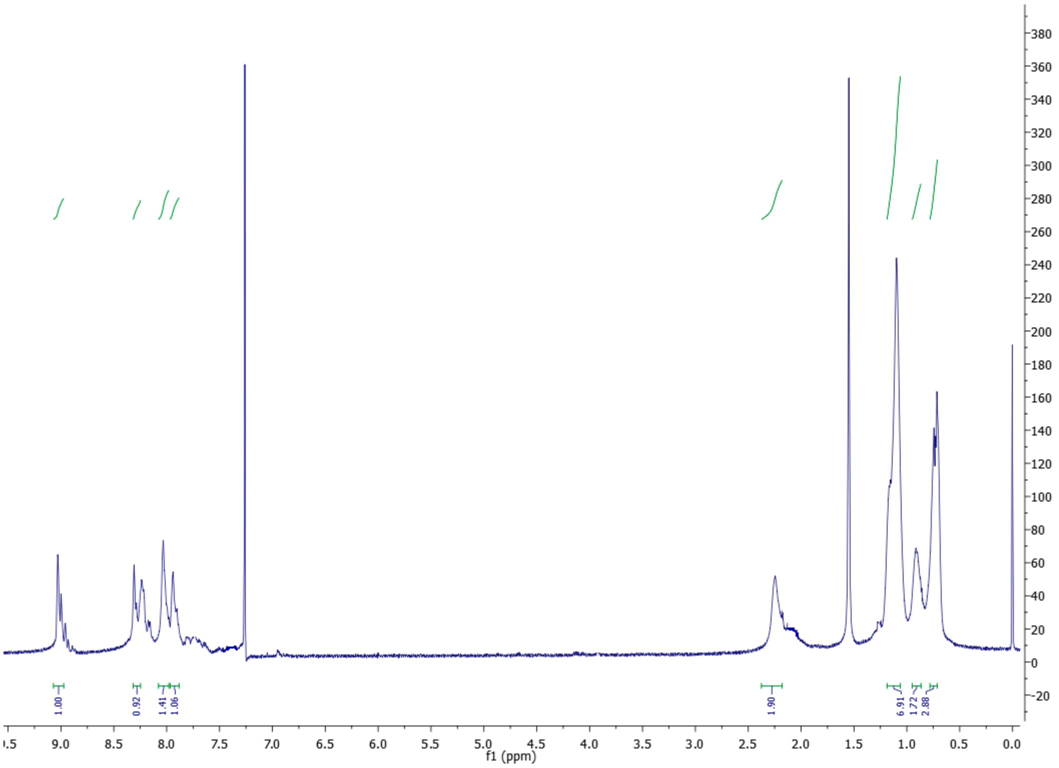

Figure S30. ^{1}H-NMR Spectra of polymer **P1c**

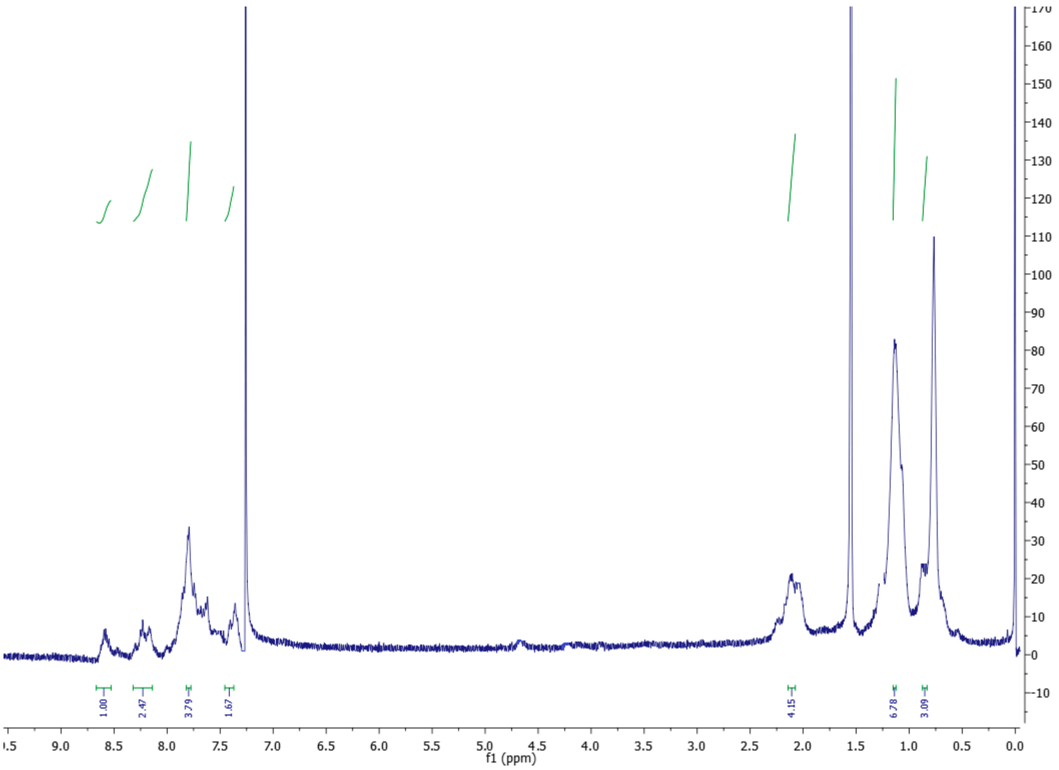

Figure S31. ^{1}H-NMR Spectra of polymer **P1d**

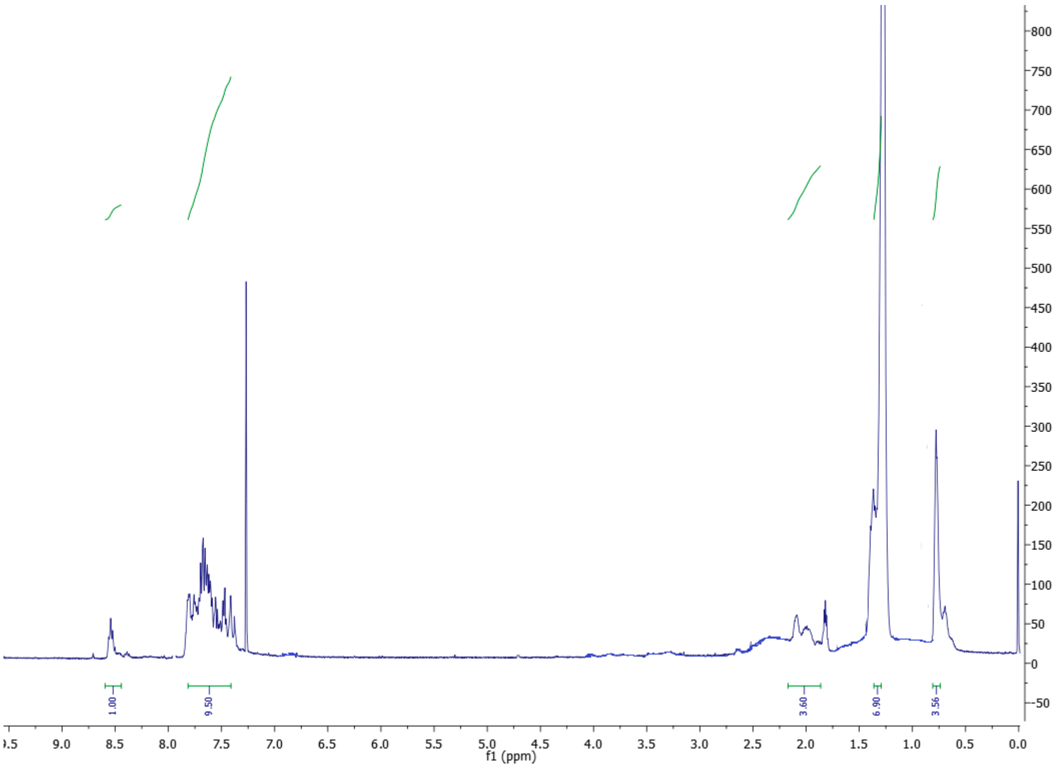

Figure S32. ^{1}H-NMR Spectra of polymer **P2b**

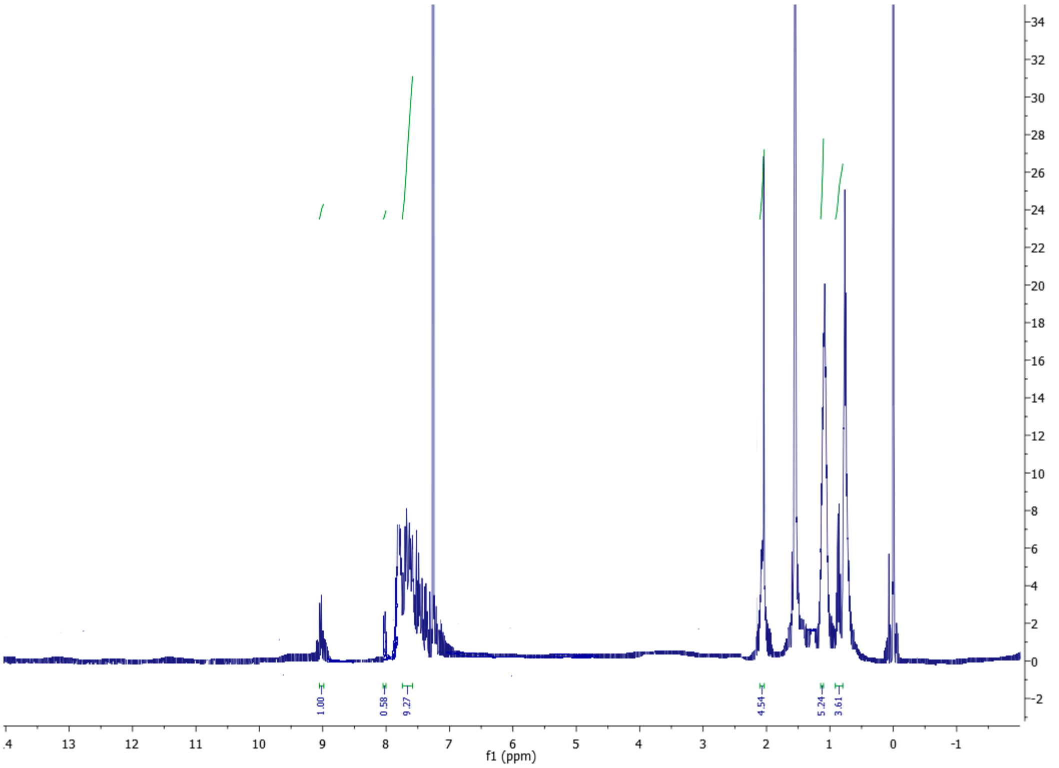

Figure S33. ^{1}H-NMR Spectra of polymer **P2c**

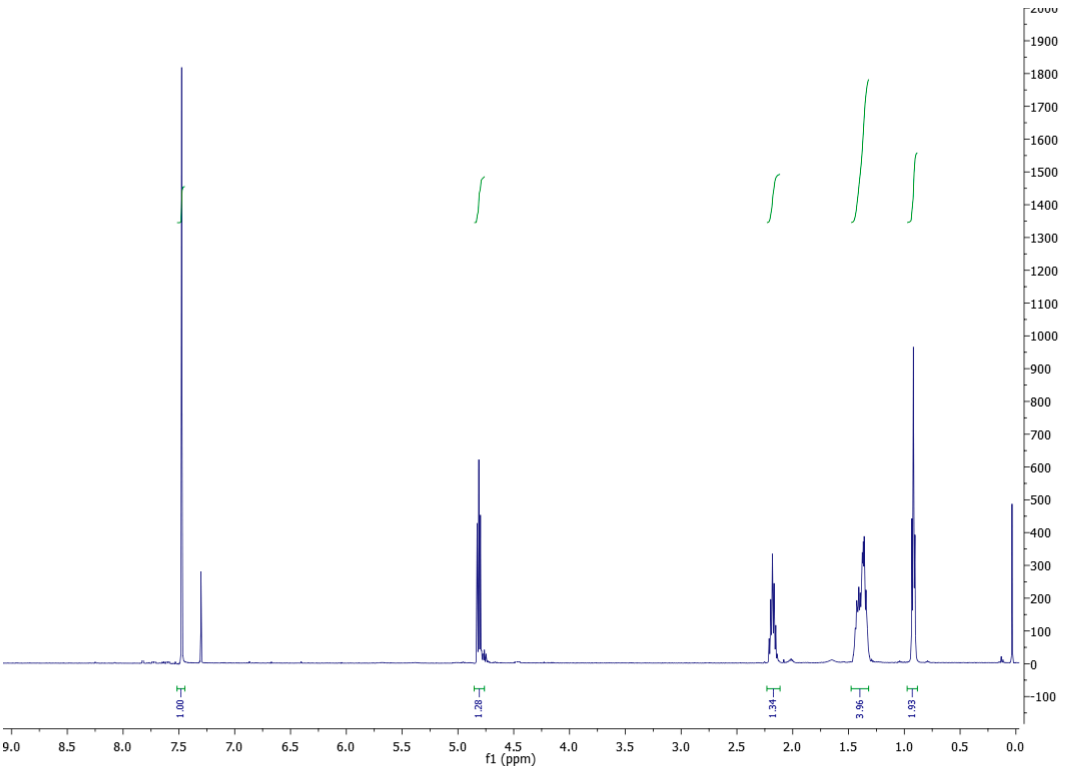

Figure S34. ^{1}H-NMR Spectra of monomer **m1a**.

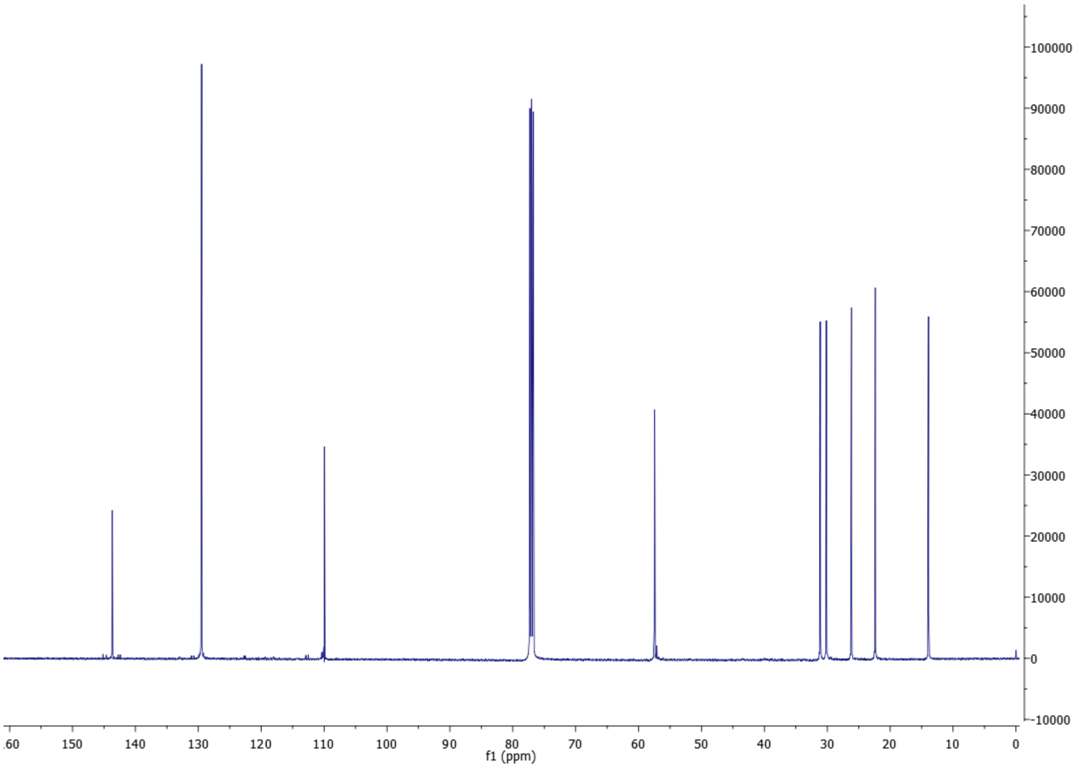

Figure S35. ^{13}C-NMR Spectra of monomer **m1a**.

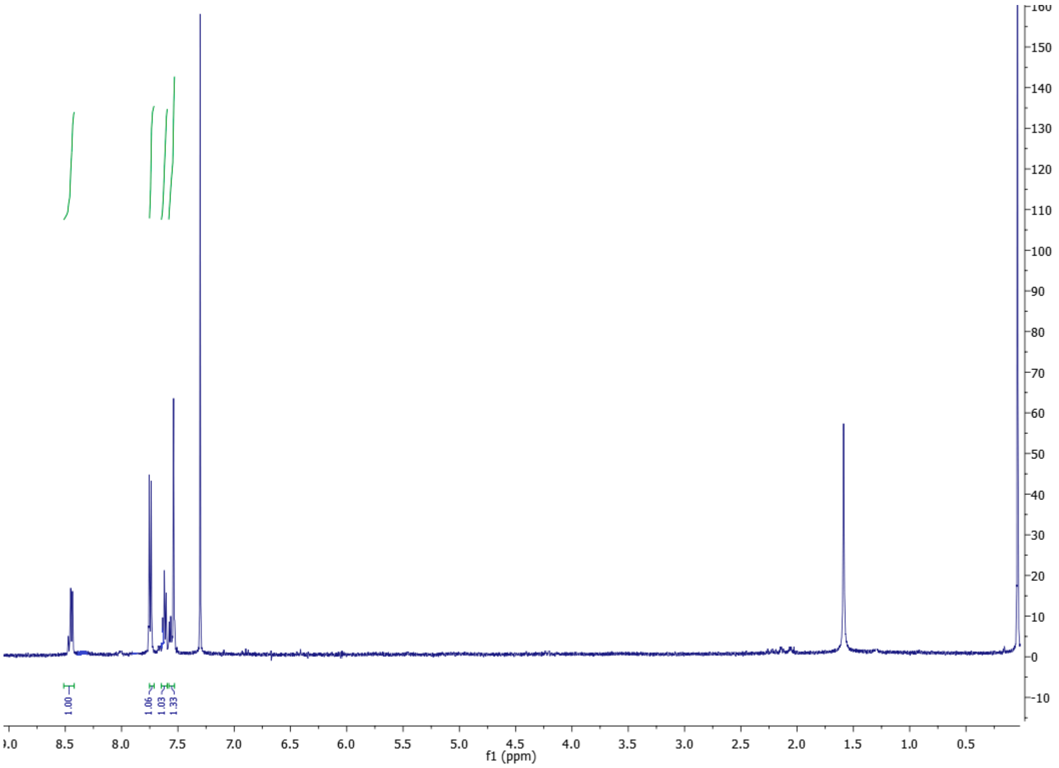

Figure S36. ^{1}H-NMR Spectra of monomer **m1b**.

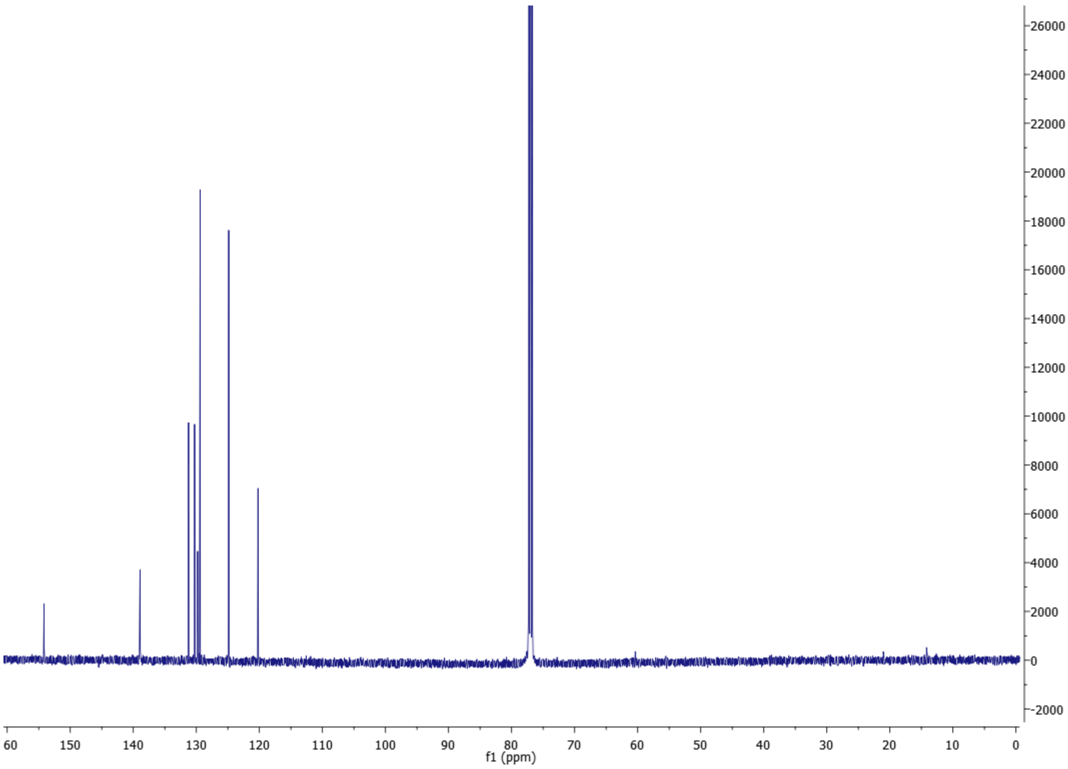

Figure S37. ^{13}C-NMR Spectra of monomer **m1b**.

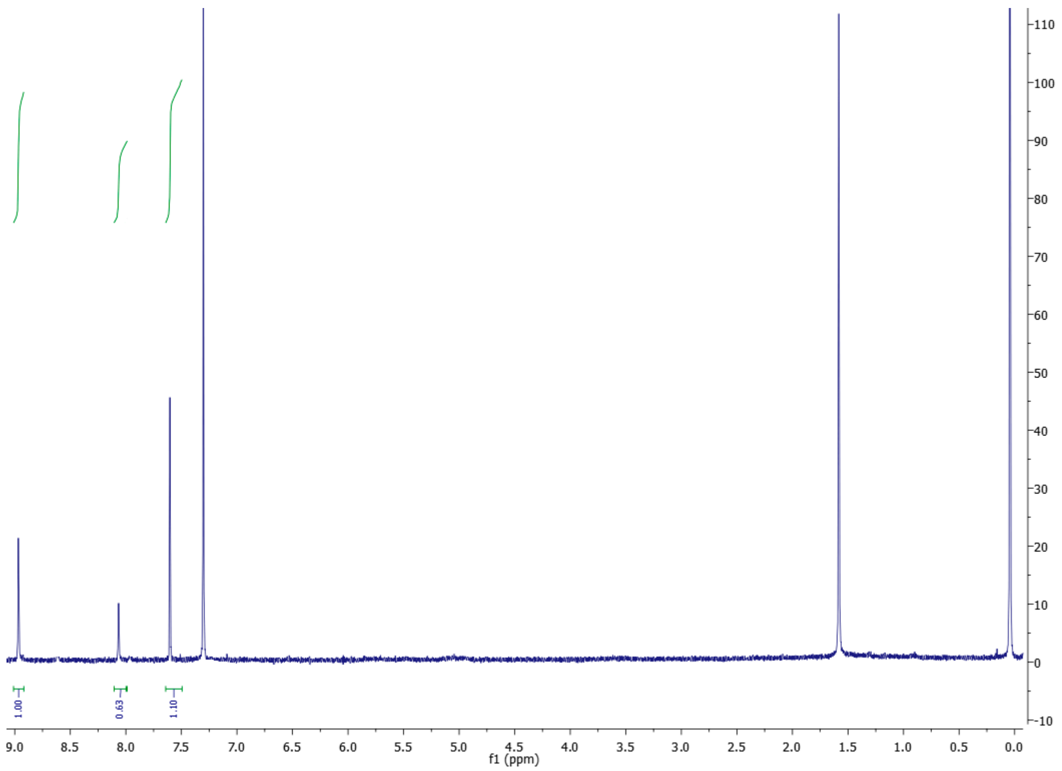

Figure S38. ^{1}H-NMR Spectra of monomer **m1c**.

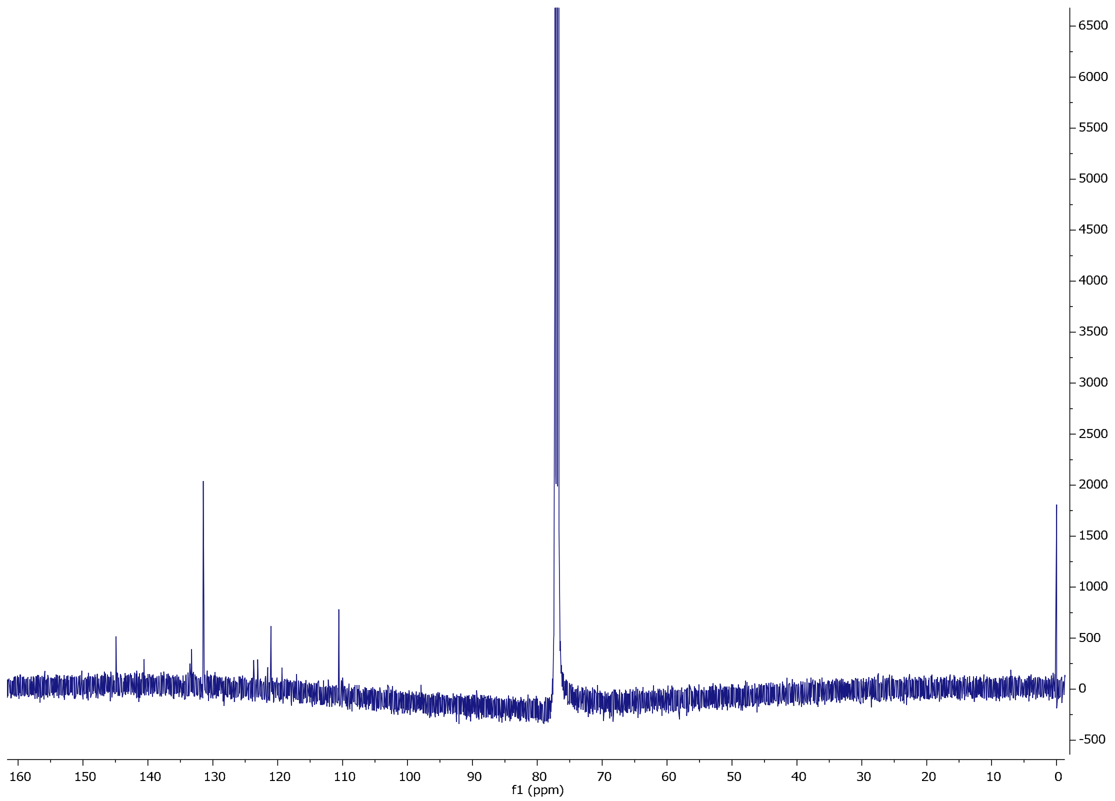

Figure S39. ^{13}C-NMR Spectra of monomer **m1c**.

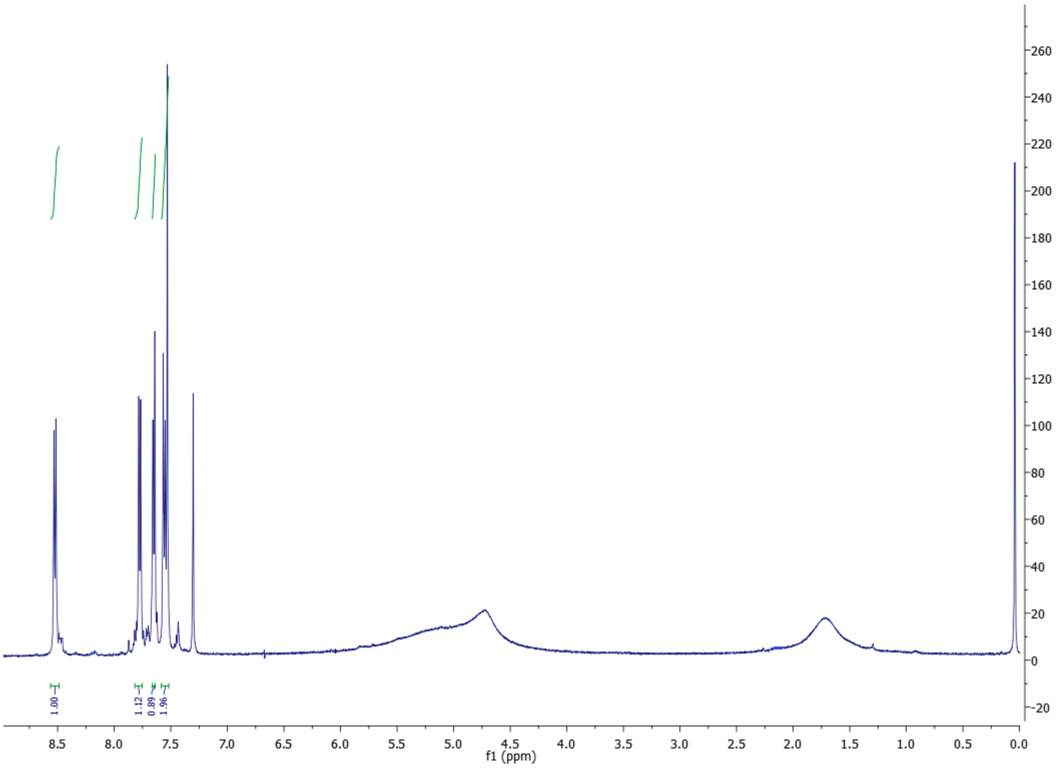

Figure S40. ^{1}H-NMR Spectra of monomer **m1d**.

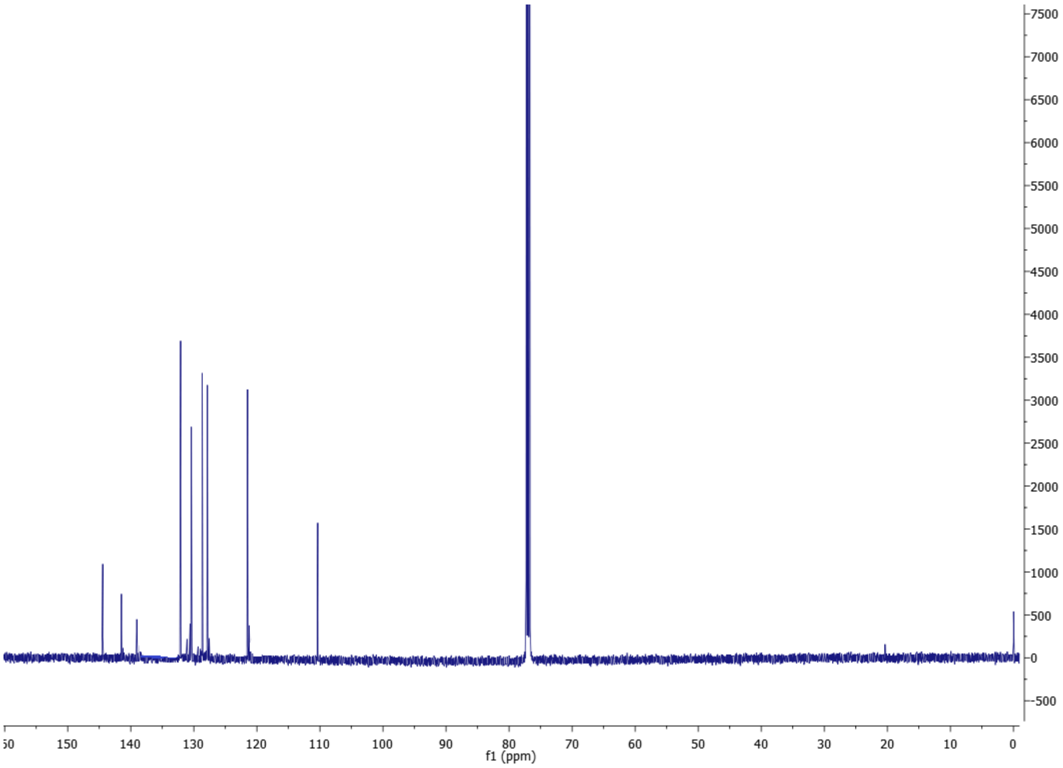

Figure S41. ^{13}C-NMR Spectra of monomer **m1d**.

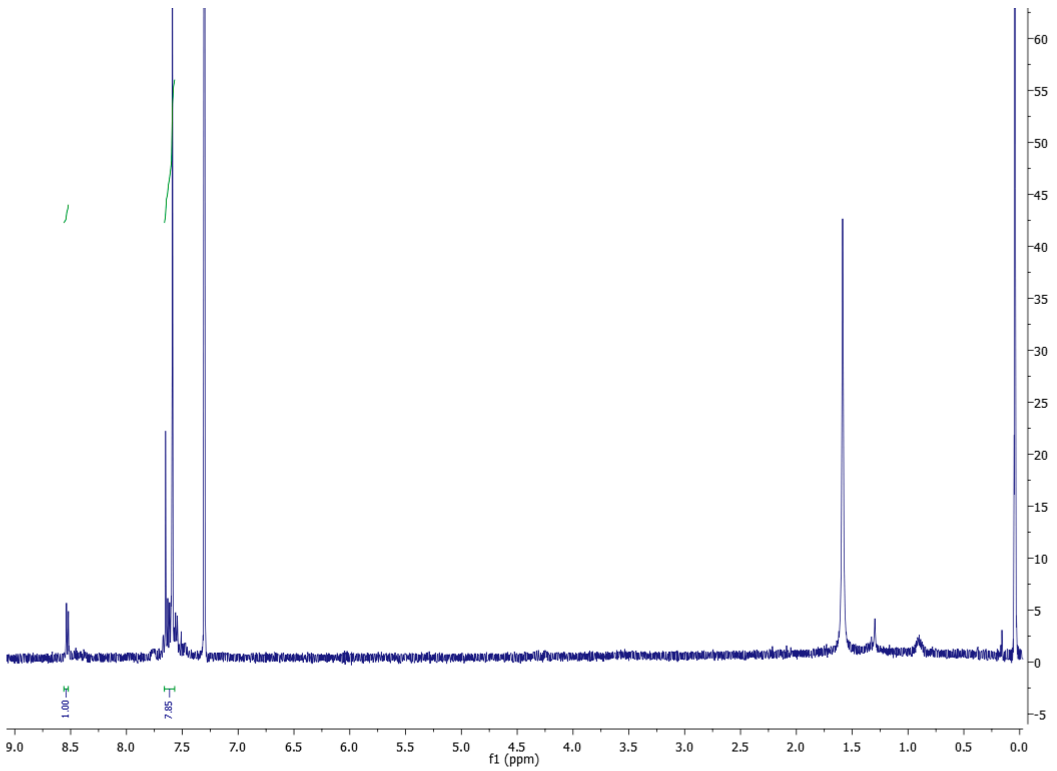

Figure S42. ^{1}H-NMR Spectra of monomer **m2b**

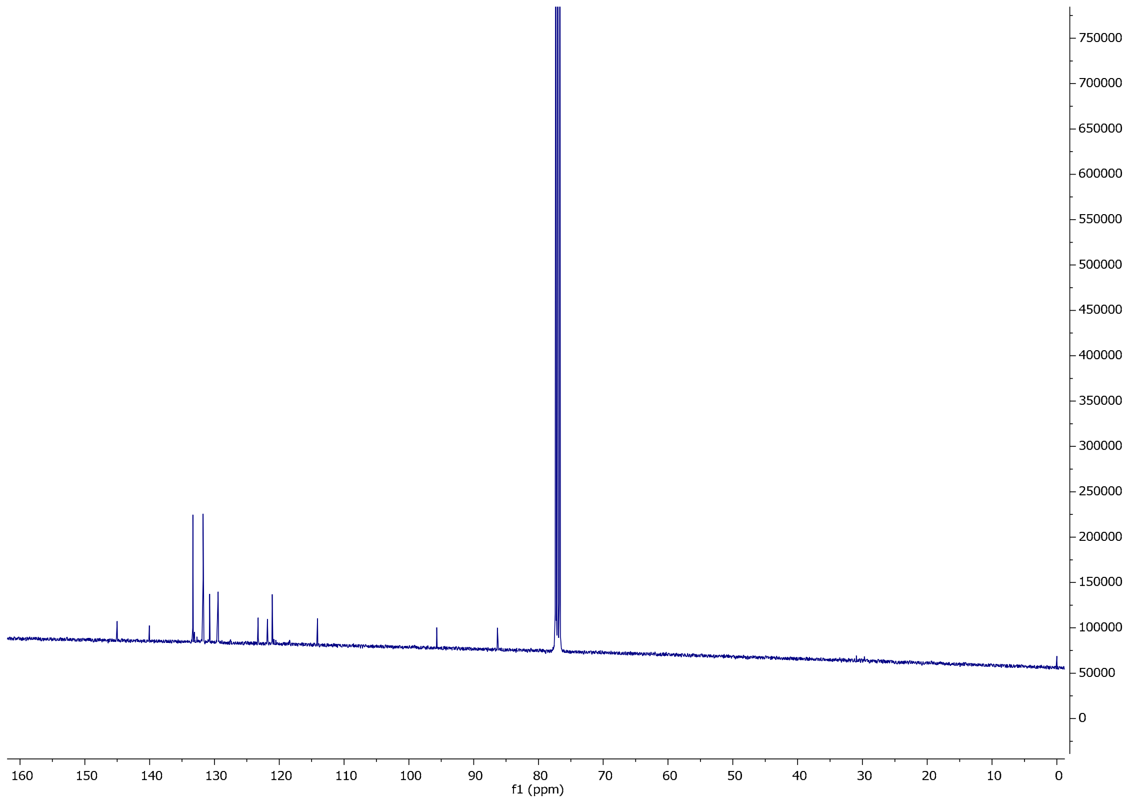

Figure S43. ^{13}C-NMR Spectra of monomer **m2b**

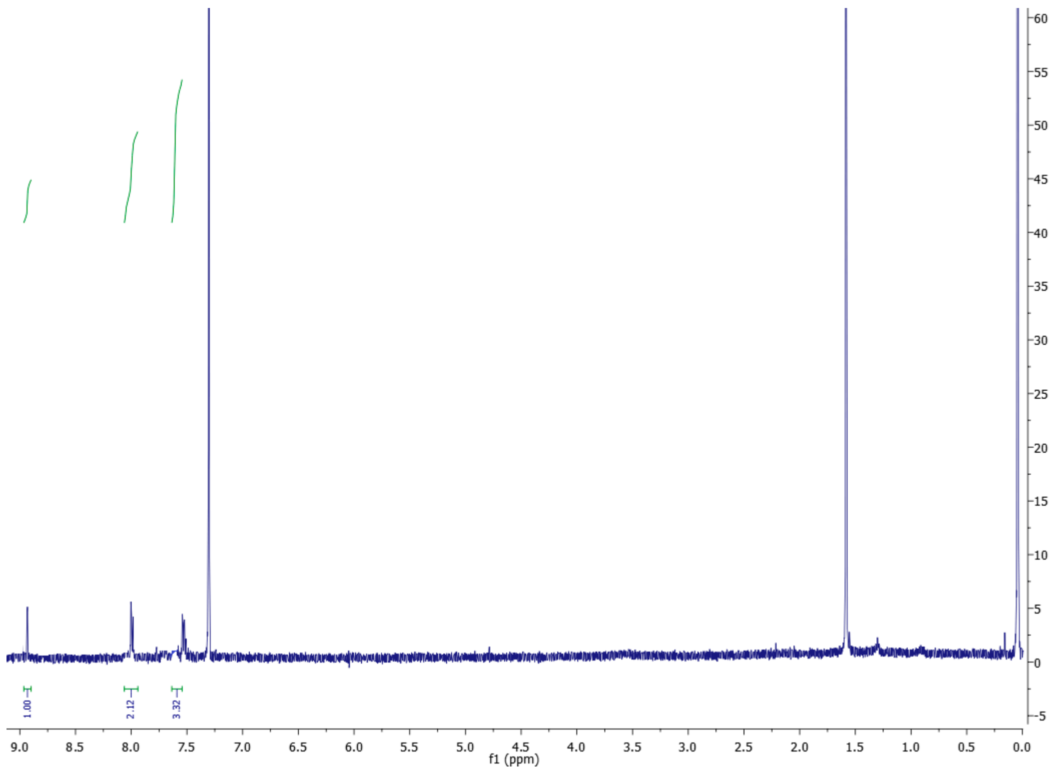

Figure S44. ^{1}H-NMR Spectra of monomer **m2c**

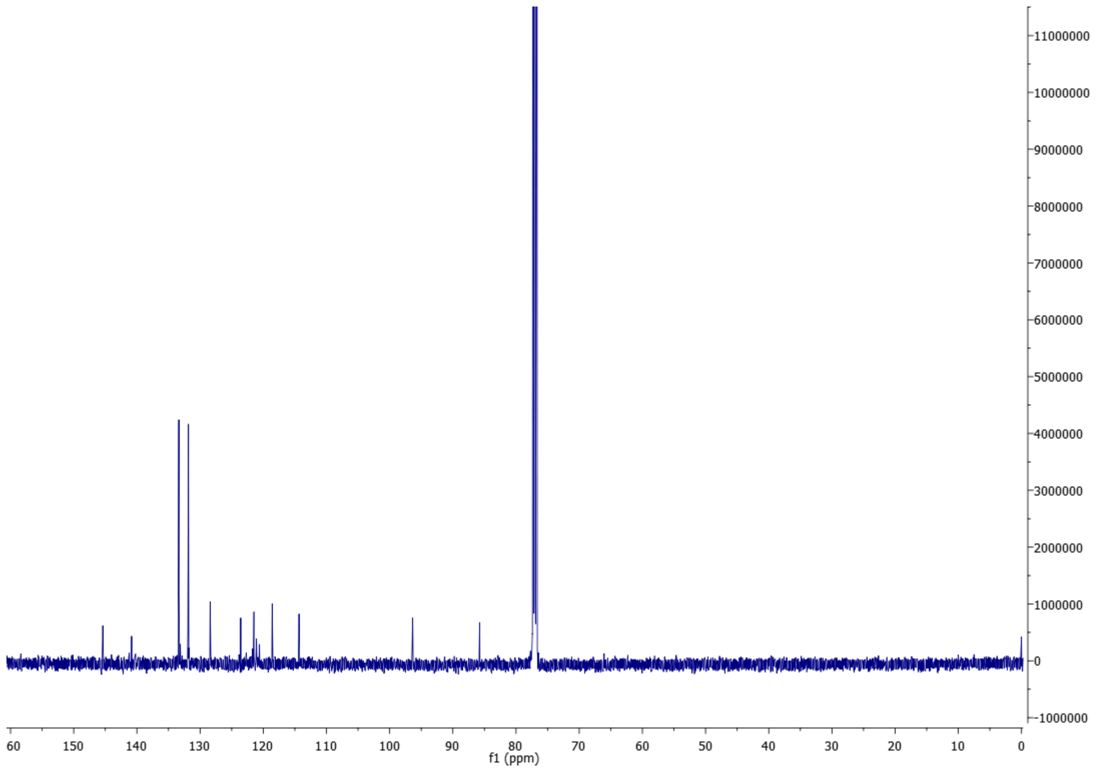

Figure S45. ^{13}C-NMR Spectra of monomer **m2c**

5. Collection of Raman spectra

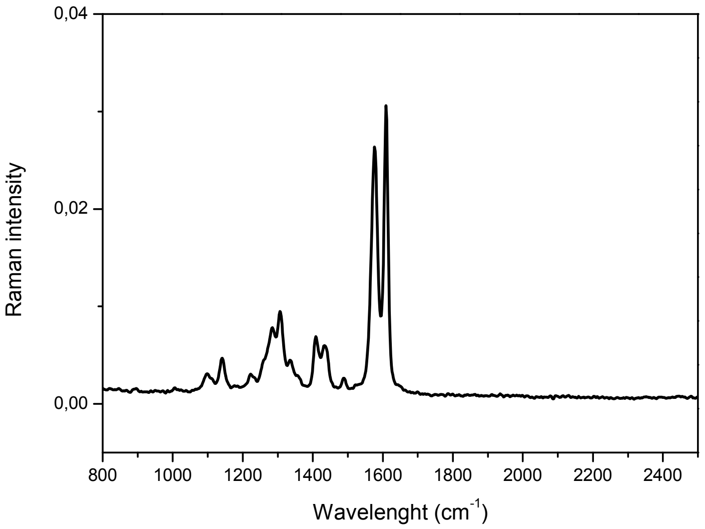

Figure S46. Raman spectrum for polymer **P1a**.

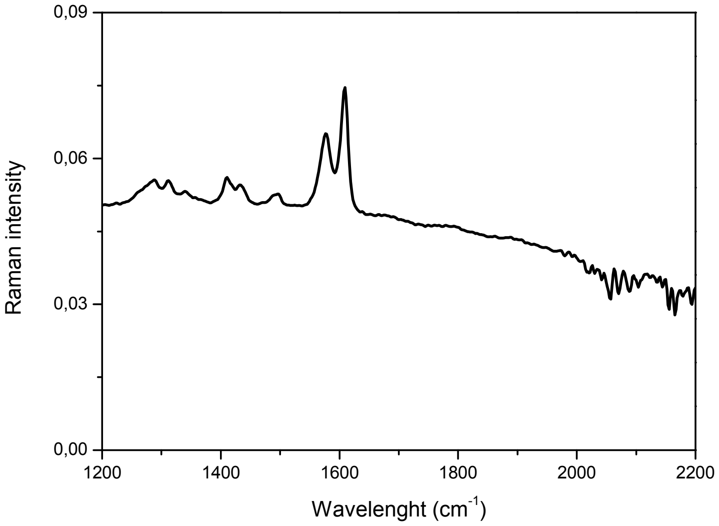

Figure S47. Raman spectrum for polymer **P1b**.

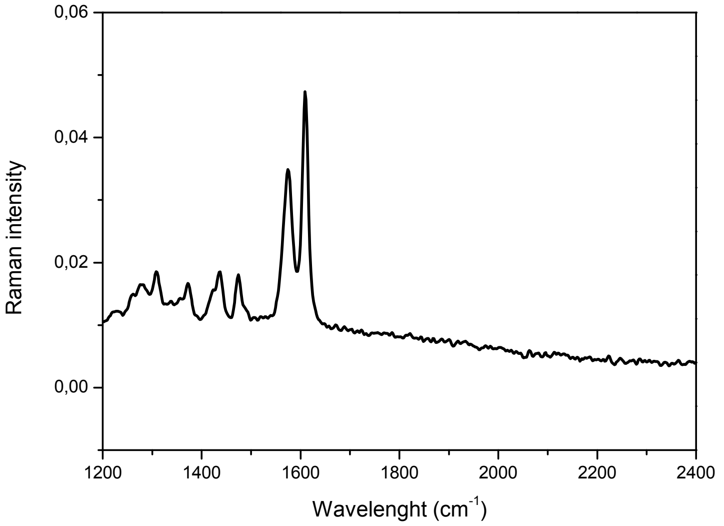

Figure S48. Raman spectrum for polymer **P1c**.

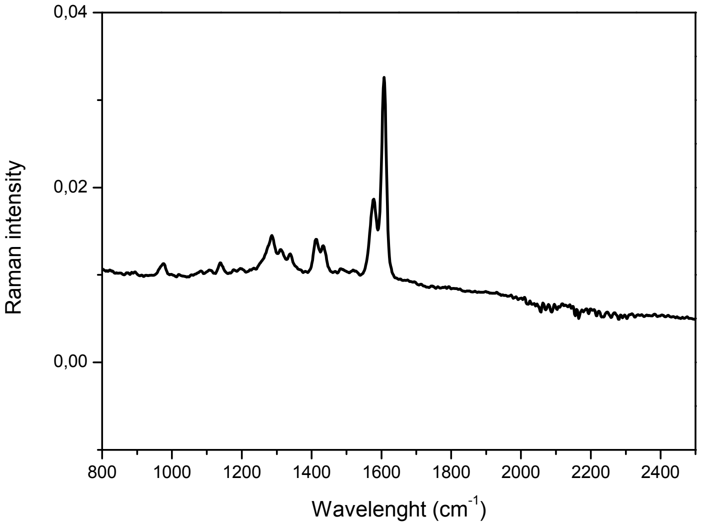

Figure S49. Raman spectrum for polymer **P1d**.

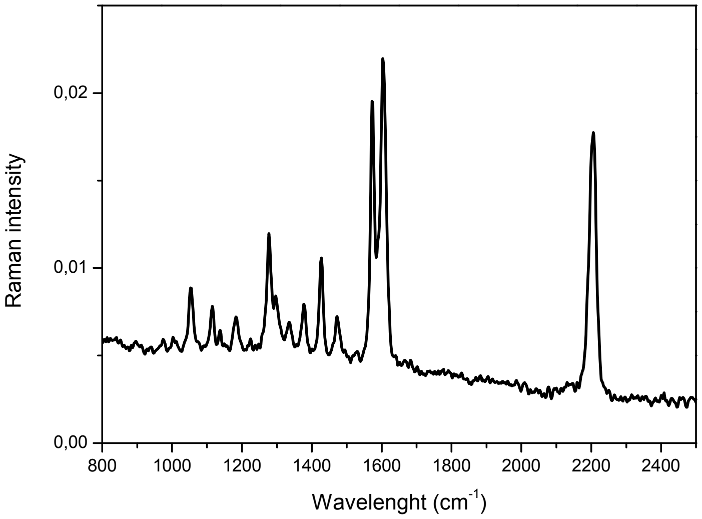

Figure S50. Raman spectrum for polymer **P2c**.
